# Societal beliefs about pain may be more balanced than previously thought. Results of the Guernsey pain survey

**DOI:** 10.1186/s12891-023-07088-0

**Published:** 2024-01-18

**Authors:** Martin Rabey, Helen Slater, Clair Hebron, Niamh Moloney

**Affiliations:** 1 Thrive Physiotherapy, St. Martin, Guernsey; 2 School of Allied Health, Curtin University, Kent St. Bentley, WA 6102 Australia; 3 School of Sport and Health Sciences, University of Brighton, Brighton, UK; 4 enAble Institute, Curtin University, Kent St. Bentley, WA 6102 Australia

**Keywords:** Pain, Societal beliefs, Perspectives, Understanding, Survey

## Abstract

**Background:**

Musculoskeletal pain is multidimensional and associated with significant societal impact. Persistent or chronic pain is a public health priority. A step towards high-value care is a contemporary understanding of pain. While pain-related knowledge has been examined in specific conditions (e.g. neck pain) knowledge of the public’s broader understanding regarding musculoskeletal pain per se*,* warrants investigation. This study examined the public’s knowledge and beliefs regarding musculoskeletal pain and pain management.

**Methods:**

This observational cohort study was conducted in Guernsey (January 2019-February 2020). Participants (*n* = 1656; 76.0% female) completed an online questionnaire capturing: demographics, pain experience, work absenteeism, understanding of pain and pain management, multidimensional influences, physical activity, pain catastrophising and healthcare decision-making. Statements were deemed true/false/equivocal and mapped to biopsychosocial/biomedical/neutral perspectives based upon contemporary literature.

Descriptive statistics were analysed for each statement. Participants’ responses were examined for alignment to a contemporary viewpoint and themes within responses derived using a semi-quantitative approach modelled on direct content analysis. Comparisons between participants with/without pain were examined (χ^2^-squared/Wilcoxon Rank Sum test).

**Results:**

Within the cohort 83.6% reported currently experiencing pain. The overarching theme was perspectives that reflected both biomedical and contemporary, multidimensional understandings of pain. Sub-themes included uncertainty about pain persistence and evidence-based means to reduce recurrence, and reliance upon healthcare professionals for guiding decision-making. Compared to those with pain, those without had a greater belief that psychological interventions may help and lower pain catastrophising.

**Conclusions:**

Participants’ understanding of pain demonstrated both biomedical and multidimensional pain understanding consistent with elements of a contemporary understanding of pain.

**Supplementary Information:**

The online version contains supplementary material available at 10.1186/s12891-023-07088-0.

## Background

Acute musculoskeletal pain is very common [1], and people follow variable trajectories from recovery through to persistent severe pain [2, 3]. A contemporary understanding of pain is of a complex multidimensional experience [4], which recognises a person’s “being in the world” with cultural, temporal, emotional and intersubjective influences including influences from significant others and wider society [5, 6]. While most acute pain resolves, persistent or chronic pain affects 35–51% of the UK population [7] and has a significant economic burden [8] and societal impact. Calls to address chronic pain at a global level [9–11] reflect chronic pain being considered a public health priority [12]. In this context, musculoskeletal pain is recognised as a leading cause of disability globally [13]. However, only half of national health policies within the Organisation for Economic Co-operation and Development have an explicit focus on prevention and management of musculoskeletal pain [14]. Where these countries focus on musculoskeletal pain, policy aims include addressing risk factors, promoting physical and social functioning and public health education to change health beliefs and facilitate positive pain-related behaviours [14]. Another common policy aim is to deliver high-value (i.e. high quality) pain care [10]. However, limited public understanding regarding pain, pain management and the experiences of those living with persistent pain, remain barriers to high-value care [15].

Clinical populations may, and often do, hold predominantly biomedical beliefs, i.e. that pain is proportional to pathology and that identifying pathology is critical to obtaining appropriate healthcare [16]. However, across the multidimensional influences on pain an individual’s pain presentation is likely to be highly variable [17, 18] even in the acute phase, with biomedical factors frequently becoming less dominant with increasing chronicity [19]. Holding dominantly biomedical beliefs as either a person/patient or clinician is associated with poorer health outcomes [20, 21], yet current evidence highlights a continued dependence on biomedical factors to explain the development and persistence of pain [22]. Such beliefs may be unhelpful and impede people’s engagement with self-management, the latter being a core component of best practice pain care [23].

Improving health literacy and engagement with health information improves autonomy and agency in healthcare [24], critical for optimising health outcomes. Part of informing high-value care is making sense of pain, and this education should align with a contemporary understanding of pain, including individualised education and self-management [15, 25].While pain-related beliefs and knowledge have been widely examined in cohorts with specific pain conditions (e.g. low back pain [26], neck/arm pain [27]), the public’s broader knowledge, understanding and beliefs regarding musculoskeletal pain per se*,* warrants investigation. Therefore, the primary aim of this study was to examine the public’s knowledge and beliefs regarding musculoskeletal pain and pain management. The secondary aim of this study was to examine differences in knowledge and beliefs between those with lived pain experience and those without.

## Methods

Human research ethics approval was granted for this study by the Guernsey Ethics Committee (July 2018 Meeting) and this research complied with the Declaration of Helsinki [28].

This was an observational cohort study. Data were collected between January 2019-February 2020 in Guernsey. Participants were recruited via print and social media, and advertising in community and clinical settings. Questionnaires were not sent to participants directly. Potential participants were directed to a custom website where they accessed participant information, gave informed consent to participate and completed an online questionnaire. Recruitment targeted those with *and without* pain conditions. Inclusion criteria were aged 18 or over and resident in Guernsey.

Sample size was estimated as follows. Based upon the results of any one question in the questionnaire, the sample size was estimated as the number of participants required to answer the question so that their result fell within 10 percentage points of the true population answer with 95% confidence. The likely responses to each individual question were assumed to be unknown. Therefore, the true population proportion with a specific answer to an individual question was assumed to be 0.5 to generate the largest sample size necessary. Therefore, the required sample size was 385 participants [29].

The questionnaire developed to explore beliefs important to the public understanding of musculoskeletal pain (Additional file) comprised 114 questions from published questionnaires [30–32] and items specifically derived from literature review and expert clinician/researcher consensus (Additional file). Questions were chosen to limit participant burden and redundancy (defined by the research team as questions exploring similar/overlapping concepts). Total time for completion of the questionnaire was approximately 20 min.

Questionnaire domains captured included:

1. Demographics: age, gender, educational level
2. Pain experience
3. Work absenteeism [33]
4. Understanding of pain and pain management: including questions from the Revised Neurophysiology of Pain Questionnaire [30] and Avoidance-Endurance Questionnaire [31]
5. Multidimensional influences on pain
6. Physical activity and pain (Taken from the Fear-Avoidance Beliefs Questionnaire) [32]
7. Pain Catastrophising Scale (PCS) [34]
8. Healthcare decision-making

Data were described using frequencies and percentages for categorical variables and median (interquartile ranges) for continuous variables. Participants responses to statements were examined using a semi-quantitative approach modelled on direct content analysis. Direct content analysis utilises pre-existing theory / evidence (e.g. that pain is multidimensional but that biomedical beliefs are common) to determine working concepts through which to analyse data. Therefore, for each statement, based upon contemporary literature, the research team determined whether the answer was true/false/equivocal and whether it mapped to biopsychosocial/biomedical/neutral perspectives on pain (Additional file). If > 60% of participants offered an answer reflecting contemporary evidence the participants were deemed in agreement with the evidence. The research team considered this cut-off to offer a level of agreement (versus 40% disagreement) that would be exploratory, reducing the risk of type II errors, although possibly increasing the risk of type I error. Direct content analysis determines themes by examining frequencies of responses in relation to working concepts based upon pre-existing theory / evidence (e.g. number of responses in agreement with contemporary evidence, number of responses reflecting biomedical viewpoints). However, examination of the data may also reveal inherent themes that were not pre-specified [35].

Data were analysed for the entire cohort, and then independently, based upon participants’ responses to a question asking whether they had current musculoskeletal pain or not. Comparisons between those with and without pain were examined using the χ^2^-squared test for categorical variables and the Wilcoxon Rank Sum test for skewed continuous variables. A Bonferroni correction was applied to all analyses necessitating *p* = < 0.0004 to be deemed significant (see Tables and Additional file).

## Results

**Fig. 1:**
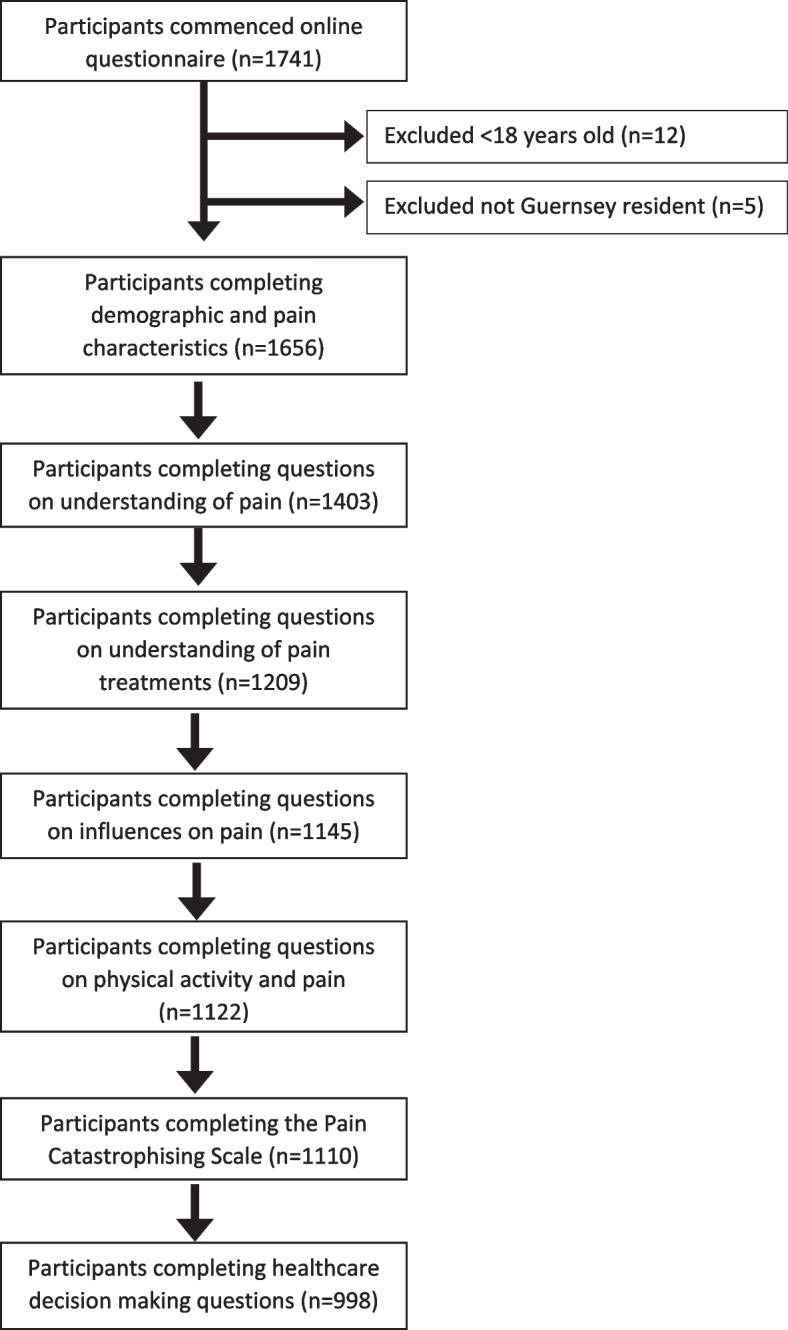
Flowchart detailing the number of participants completing each section of the questionnaire

A flowchart detailing participants completing each section of the questionnaire is given in Fig. 1.

Demographic and pain characteristic data are given for the full cohort (*n* = 1656, 2.71% of Guernsey’s population), those with (*n* = 1384, 83.6%) and without pain (*n* = 272, 16.4%) (Table 1). For the full cohort *n* = 1258 (76.0%) were female, *n* = 1384 (83.6%) were experiencing pain when completing the survey, *n* = 1083 (65.4%) had experienced pain persisting for at least three months during the previous year, of whom *n* = 652 (60.6%) described significant impact upon daily activities. Despite this, most described taking little time off work because of pain. Within the sample *n* = 97 (5.9%) had been off work because of pain for > 12-months. Those with pain were significantly older and had lower education levels than those without. Results for each individual question are given in the online Additional file.

**Table 1:** Respondents’ demographic and pain characteristic data. Data are presented as n (%)

Demographics		Full cohort (*n* = 1656)	Pain subgroup (*n* = 1384)	No pain subgroup (*n* = 272)	*p*-value*
Age (years)	18–24	178 (10.8)	129 (9.3)	49 (18.0)	** < .0001**
	25–34	271 (16.4)	208 (15.0)	63 (23.2)	
	35–44	339 (20.5)	278 (19.8)	65 (23.9)	
	45–54	360 (21.7)	316 (22.8)	44 (16.2)	
	55–64	302 (18.2)	217 (19.6)	31 (11.4)	
	65 +	206 (12.4)	186 (13.4)	20 (7.4)	
Gender	Male	393 (23.7)	323 (23.3)	70 (25.7)	.44
	Female	1258 (76.0)	1056 (76.3)	202 (74.3)	
	Other	5 (0.3)	5 (0.4)	0 (0.0)	
Highest education level completed	Primary school	9 (0.5)	7 (0.5)	2 (0.7)	**.0002**
	Secondary / high school	678 (40.9)	590 (42.6)	88 (32.4)	
	Vocational / trade qualification	469 (28.3)	396 (28.6)	73 (26.8)	
	University degree	337 (20.4)	272 (19.6)	65 (23.9)	
	Postgraduate degree	163 (9.8)	119 (8.6)	44 (16.2)	
Pain Characteristics					
Current musculoskeletal pain		1384 (83.6)	1384 (83.6)	272 (16.4)	n/a
Pain of three month’s duration ever experienced		1288 (77.8)	1178 (85.1)	110 (40.4)	** < .0001**
Pain of three month’s duration during last year		1083 (65.4)	1045 (75.5)	38 (14.0)	** < .0001**
If pain of three month’s duration during last year, pain interference in past week^a^	None at all	63 (5.8)	50 (4.8)	13 (34.2)	** < .0001**
	A little	362 (33.6)	350 (33.7)	12 (31.6)	
	Quite a bit	353 (32.8)	347 (33.4)	6 (15.8)	
	A lot	299 (27.8)	292 (28.1)	7 (18.4)	
Days off work because of pain during last year	0 days	1008 (61.6)	795 (58.1)	213 (79.5)	** < .0001**
	1–2	132 (8.1)	116 (8.5)	16 (6.0)	
	3–7	139 (8.5)	119 (8.7)	20 (7.5)	
	8–14	79 (4.8)	73 (5.3)	6 (2.2)	
	15–30	68 (4.2)	67 (4.9)	1 (0.4)	
	1–3 months	59 (3.6)	51 (3.7)	8 (3.0)	
	4–6	34 (2.1)	32 (2.3)	2 (0.8)	
	7–12	20 (1.2)	20 (1.5)	0 (0.0)	
	> 12	97 (5.9)	95 (6.9)	2 (0.8)	

^a^*n* = 1077

^b^*n* = 1636

^*^p-value reflects difference between pain and no pain subgroups

One overarching theme was derived from the entire cohort data: complex/conflicting perspectives across biomedical and contemporary, multidimensional understandings of pain and pain care. Table 2 gives key statements reflecting participant alignment with both biomedical and multidimensional understandings of pain, e.g. “Pain may mean something is out of place,” (76.0% agreement) versus, “There is always tissue damage to explain pain,” (71% disagreement).

**Table 2:** Diverse / conflicting perspectives: Biomedical or multidimensional understandings of pain. Key statements

Biomedical			Multidimensional		
“Pain may mean something is out of place”		76.0% agreement	“Pain only occurs when you are injured or at risk of being injured”		85.1% disagreement
“When you injure yourself, the environment that you are in will not affect the amount of pain you experience, as long as the injury is exactly the same”		64.9% disagreement	“There is always tissue damage to explain pain”		71.0% disagreement
“Exercise is always helpful for treating pain”		69.1% disagreement	“There is always a simple explanation for why someone has pain”		83% disagreement
“You should be very careful exercising when you have pain”		90.2% agreement	“Pain means you aren’t healthy”		87.6% disagreement
“Good core stability is key to managing pain”		19.3% disagreement	“It is important to stay active when you have pain”		63.8% agreement
“It is always important to maintain good alignment when exercising, especially if you have pain”		85.8% agreement	“It is important to gradually increase your activity when you have pain”		67.7% agreement
Participants’ perceived influences on pain	Posture and alignment	91.5% agreement	“It is possible to manage pain well yourself”		68.2% agreement
	Amount of tissue damage or injury	91.4% agreement	“Understanding how pain works is an effective pain treatment”		63.9% agreement
	Weight	85.9% agreement	“It is important to treat underlying lifestyle factors for pain relief (e.g. sleep, stress, work habits, exercise, diet)”		89.7% agreement
	Muscle tightness	80.8% agreement	“Psychological treatments (talk therapies, stress management, mindfulness) can be helpful for treating pain”		69.1% agreement
	Muscle weakness	79.9% agreement	“Surgery should only be considered as a final option when other treatments have not worked”		76.7% agreement
	Education level	10.4% agreement	“Exercise can be helpful for treating pain”		91.2% agreement
	Sex	18.2% agreement	Participants’ perceived influences on pain	Sleep	71.7% agreement
	Culture	23.4% agreement		Stress	71.6% agreement
	Social support	28.7% agreement		Comorbidities	63.8% agreement
				Mood	61.6% agreement

^a^Participants could be in agreement or disagreement with each statement. Dependent upon the statement agreement or disagreement may reflect a biomedical view e.g. for the statement, “Pain may mean something is out of place,” agreement would suggest a biomedical viewpoint, while for the statement, “When you injure yourself, the environment that you are in will not affect the amount of pain you experience, as long as the injury is exactly the same,” disagreement would suggest a biomedical viewpoint. Multidimensional viewpoints could be reflected in a similar manner

Sub-themes derived include uncertainty about prognosis in pain disorders (see Table 3 for key statements) and a reliance, predominantly, upon healthcare professionals for guiding healthcare decision-making (see Table 4 for key statements).

**Table 3:** Uncertainty about prognosis regarding prognosis in musculoskeletal pain: Key statements

“Once you have pain you’re always likely to have pain”	71.7% disagreement
“Most pain gets better”	36.6% agreement, 44.8% disagreement, 18.5% unsure
“Future episodes of pain cannot be avoided”	41.0% agreement, 38.0% disagreement, 21% unsure
“Future episodes of pain can be reduced or avoided by avoiding aggravating activities”	77.2% agreement
“Future episodes of pain can be reduced or avoided by addressing lifestyle factors like sleep, weight and stress”	80.1% agreement

**Table 4:** Dominance of healthcare professionals for providing healthcare guidance for pain care: Key statements

“It is important to seek professional advice for pain care”		71.7% agreement
“It is important to seek treatments (medications, injections, surgery, hands-on treatments) from professionals to get pain relief”		61.0% agreement
“What influences your decisions to have certain types of treatments?”	Highest ranking influence: General Practitioner	44.7%
	Highest ranking influence: Other healthcare professional	38.2%
	Highest ranking influence: Self-care	9.2%
	Decision-making not influenced by healthcare professionals	24.5%
	Highest ranking influence: Internet	6.0%
	Scientific evidence not considered	24.8%

### Discordance between participants’ responses and a contemporary understanding of pain

There were 92 statements about pain beliefs and its management requiring a true/false/equivocal answer of which 10 were deemed equivocal by the research team. Therefore, agreement between participants and the research team was based upon results from 82 statements. Participants agreed with researchers for 53 statements (64.6%), disagreed for 11 statements (13.4%) and were unsure (< 60% agreement) for 18 statements (22.0%) (Table 5).

**Table 5:** Participants’ agreement with statements deemed true, false or equivocal by the research team

Statements where participants disagreed with the research team			Statements where participants’ responses were unsure		Statements deemed equivocal by the research team		
Statement	Research team’s response	Participants’ response	Statement	Research team’s response	Statement	Research team’s answer	Participants’ answer
Pain may mean something is out of place	False, Med	76.0% True	Most pain gets better	True, N	Persistent pain means that an injury hasn’t healed properly	Med	Unsure
When I have pain I think to myself “don’t make such a fuss”	False, N	77.0% True	More pain means more tissue damage (i.e., damage to joints, nerves, tendons or muscles)	False, Med	An increase in pain is an indication that you should stop doing what you’re doing until the pain decreases	Med	Unsure
It is important to seek treatments (medications, injections, surgery, hands-on treatments) from professionals to get pain relief	False, N	61.0% True	Findings on scans like arthritis and disc bulges are always associated with pain	False, Med	It is important to rest when you have pain	Med	Unsure
Surgery should only be considered as a final option when other treatments have not worked	False, Med	76.7% True	Tests like MRI scans, x-rays and ultrasound imaging are critical to identify the source of pain	False, Med	It is important to seek professional advice for pain care	N	71.7% True
It is always important to maintain good alignment when exercising, especially if you have pain	False, Med	85.8% True	The source of pain must always be identified for adequate pain treatment to occur	False, Med	You should be very careful exercising when you have pain	Med	90.2% True
Future episodes of pain can be reduced or avoided by avoiding aggravating activities	False, Med	77.2% True	When I have pain I carry on doing what I’m doing no matter what	False, N	Understanding how pain works is an effective pain treatment	N	63.9% True
Influencing factor: Muscle tightness	False, Med	80.8% True	Stretching is always an effective exercise for pain	False, Med	I am usually willing to change my habits and behaviours to improve my health and pain care	BPS	88.0% True
Influencing factor: Social support	True, BPS	28.7% True	Good core stability is key to managing pain	False, Med	Future episodes of pain can be reduced or avoided by addressing lifestyle factors like sleep, weight and stress	BPS	80.1% True
Influencing factor: Culture	True, BPS	23.4% True	Good advice can be sufficient pain care	True, N	Influential factor? Posture and alignment (e.g., spinal posture, leg alignment, foot posture)	Med	91.5% True
Influencing factor: Sex/Gender	True, Med	18.2% True	Physical therapies (physiotherapy, osteopathy, chiropractic) should always include ‘hands-on’ treatments for pain relief	False, Med	Influential factor? Ergonomics (e.g., work set up and practices)	Med	66.7% True
Influencing factor: Education level	True, BPS	10.4% True	Future episodes of pain can be reduced or avoided through exercise	True, N			
			Future episodes of pain can be reduced or avoided by getting regular ‘hands-on’ treatments like massage or manipulation	False, Med			
			Future episodes of pain cannot be avoided	True, N			
			Influencing factor: Beliefs about injury and tissue damage	True, BPS			
			Influencing factor: How you think about pain	True, BPS			
			Influencing factor: Access to appropriate healthcare	True, N			
			Influencing factor: Alcohol or drug use	True, Med			
			Influencing factor: Genetics	True, Med			

Med: medical; BPS: biopsychosocial; N: neutral

The research team deemed statements reflective of biomedical (Med), biopsychosocial (BPS) or neutral/neither (N) viewpoints

### Comparison of those with and without pain

When comparing participants’ understanding of pain there were several significant differences between those with and without pain (Bonferroni correction *p* = < 0.0001).Only 33.9% of those with pain thought, “Most pain gets better,” was a true statement, versus 51.4% with no pain; while 62.6% of those with pain agreed with the statement, “When I have pain, I carry on doing what I am doing no matter what,” compared to 43.6% with no pain.

When comparing participants’ understanding of pain treatments the only significant between group difference regarding treatment efficacy was 80.8% of those without pain believed psychological treatments can be helpful for pain compared to 66.9% of those with pain. The belief that future pain episodes cannot be avoided was more common in those with pain (43.8%) than without (25.7%).

When comparing participants’ perceived influences on pain the only significant difference between groups was that those without pain agreed that beliefs about injuries/tissue damage could influence pain more often than those with pain (55.4% versus 39.3%).

When examining participants’ beliefs regarding physical activity and pain [8] there were no significant differences.

Regarding pain catastrophising those with no pain were less likely to agree with statements, “When I’m in pain…. It’s terrible and I think it’s never going to get any better; It’s awful and I feel that it overwhelms me; I feel I can’t stand it anymore; I become afraid that the pain will get worse.” The first three statements are from the helplessness subscale of the PCS [36]. There were significant between group differences (median scores; pain versus no pain) for the helplessness subscale (7-points versus 4-points), total PCS score (17-points versus 12-points) and proportion of participants scoring > 30-points on the PCS (21.2% versus 8.3%).

When considering participants’ healthcare decision-making there were no significant differences.

## Discussion

This research demonstrates a novel exploration of the multidimensional understanding of pain at a societal level, including people with and without pain. It included a high proportion of participants with self-reported current musculoskeletal pain. Participants’ understanding of pain demonstrated both biomedical beliefs (i.e. regarding the relationship between pain and tissue pathology) and multidimensional pain understanding (i.e. pain is influenced by broader health and emotional well-being) consistent with elements of a contemporary understanding of pain and pain management. Of 11 statements for which there was discordance between participants’ answers and a contemporary viewpoint, eight suggested dominance of biomedical beliefs. Some conflict between biomedical and multidimensional views may reflect perceived context (e.g. acute tissue injury v. chronic non-specific low back pain) not captured in this questionnaire (See unsure/equivocal sections, Table 5), or indeed paradoxical beliefs reflecting a complex adaptive system [37]. Statements examining beliefs about pain care suggested a contemporary understanding of pain management. However, there was uncertainty about pain persistence and whether future episodes can be avoided. Activity limitation and avoidance of provocative activities (which is potentially harmful [38]). was the most strongly endorsed approach to prevention (77%). Further, while exercise significantly reduces pain recurrence [39], only 58% of participants endorsed exercise as important for reduction of pain recurrences similar to manual therapy, which has no known preventative effects [40]. Finally, healthcare decision-making was predominantly driven by consultations with healthcare professionals, with few seeing self-care as their chosen option to manage pain.

### Diverse / conflicting perspectives

Biomedical beliefs were evident but inconsistently so. While many participants demonstrated beliefs aligned with a biomedical/structural understanding of pain (eg. pain caused by something being out of place/poor alignment/amount of tissue damage in the absence of an influence from the environment pain experienced within), it was commonly perceived that there was not a simple explanation for pain and that pain was not necessarily associated with tissue damage. Participants were unsure regarding the need for imaging to determine a “source” of pain.

Participants biomedically-orientated beliefs may constitute overestimation of the contribution of these factors compared with research evidence. For example, research suggests the association between posture or tissue damage and pain is more variable than was considered by respondents [41, 42]. Participants’ answers may also have reflected their individual experiences and related contextual factors based on their experience of specific pathology, settings, trauma, disease, social factors, and others not captured by the current questionnaire. The relationship between pain and posture or pathology may be relevant for some individuals, [43, 44] or more evident in populations with multi-level spinal pathology [45, 46], severe osteoarthritis [47] and nerve root compression [45]. However, broad population data show correlations between posture or pathology and musculoskeletal pain are weak [48, 49] especially when psychosocial factors are considered. For example, while muscle weakness may be evident in rotator cuff-related shoulder pain [50] weakness is commonly not associated with pain when biopsychosocial factors are considered [7, 50–52]. Further, in knee osteoarthritis, interactions between physical impairments such as muscle weakness and psychological factors highlight the complex, biopsychosocial nature of pain is often underappreciated [53].

Many participants believed pain was not abnormal. This aligns with previous reports [54], and when combined with the belief that pain can occur without injury (although participant beliefs regarding “injury” were not examined), possibly suggests emergence of a contemporary multidimensional understanding of pain. Further evidence supporting a multidimensional viewpoint includes participants’ appreciation of broader health and psychological influential factors (sleep, stress, comorbidities, mood, genetics, substance use) and endorsement of a broad range of contemporary pain care strategies (staying active, graded activity, self-management, pain education, psychotherapy, behavioural change, lifestyle management) that can target individuals’ goals, needs and factors contributing to their pain. Nonetheless, that influential factors such as mood were highly ranked by only 62% of participants indicates a lower appreciation of the role of psychological factors compared to what the evidence supports [17, 55]. It is also interesting that pain-related cognitions were less frequently endorsed (42–53%) as influencing pain despite evidence to the contrary [26, 52, 56–58]. Social factors were among the lowest-ranked influences which is perhaps not surprising given the link between musculoskeletal pain and these factors may not be readily apparent to the public. However, the discordance with research findings is notable [59–62]. Together these findings suggest the public has a limited understanding of the multidimensionality of individual people’s pain experience. This could be a target of public education campaign and there is some evidence that such campaigns can be effective in shifting the public’s understanding [63].

Conflicting beliefs about pain and its influences are novel findings as previous research has largely reported the dominance of biomedical beliefs and their negative consequences [20–22, 64, 65]. This study provides findings that suggest greater nuance, reflecting public awareness of the multidimensional complexity of pain alongside biomedical beliefs. This is consistent with a complex adaptive systems perspective which acknowledges and promotes acceptance of these paradoxes. Advocates of this system propose that when accepted, greater information exchange and knowledge creation can occur versus seeking to ‘correct faulty beliefs’ [37]. While this sample had a high incidence of participants whose pain experiences may have led to a more multidimensional understanding than a sample with fewer people with persistent pain, it may be worth considering public health strategies and healthcare practitioner training to facilitate understanding of complex beliefs and a multidimensional understanding of pain in the public and clinical populations alike.

Beliefs about pain care also reflected diverse views. As well as endorsement of the contemporary pain care outlined previously, most respondents appreciated different interventions (exercise, medication, psychotherapy, injections, surgery, manual therapy) can be helpful, but are not always so, potentially reflecting an understanding that care needs to be individualised. For example, while 93% of participants stated that manual therapy can be helpful, responses were equivocal as to whether physical therapies should always include manual therapy with two thirds of respondents indicating no or unsure for this item. It is notable that nearly 70% of participants responded that psychological therapies can be helpful and should not just be used when other interventions have failed. This may provide reassurance to clinicians who may be hesitant discussing psychosocial influences impacting pain experiences in their pain care [66]. Surgery was predominantly seen as indicated when other strategies have failed, which may be helpful in the context of some conditions e.g., joint arthroplasty for osteoarthritis [67, 68]. However, individuals are likely to need counselling as to when surgery would be more clearly indicated.

Exercise was appreciated by most as important as a component of pain care consistent with evidence that indicates the role for activity/movement and exercise in pain management [69–72]; however, related responses again reflected diverse views. Most participants reported needing to “be careful” when exercising while in pain and almost 62% of participants thought the statement *“Exercise is always helpful for treating pain”* was false*.* This may reflect individual responses to exercise with some experiencing increased pain following exercise [73–75]. Furthermore, several predominantly biomedical/structurally-orientated beliefs regarding exercise (e.g. the need for ‘core stability’ or ‘good alignment’) were apparent, for which there is not compelling evidence [42, 76]. While over three-quarters of participants indicated that full pain relief was not necessary before returning to work/sport there was a broad range of responses to whether it was appropriate to rest, stay active or gradually increase activity when in pain, possibly reflecting individual experience / context (e.g. acute vs. chronic). Overall, clinicians can be encouraged that exercise is largely considered appropriate in pain care, but hesitation about engagement in exercise during pain, uncertainty about rest/activity levels and potentially unhelpful focuses on alignment and core stability suggest individuals need appropriate guidance. Hence, clinicians need to provide guidance about exercise, activity engagement and functional restoration.

### Uncertainty about prognosis

Participant responses also highlighted uncertainty about pain persistence and whether future episodes can be avoided, possibly in keeping with the prognosis of pain disorders [77–80] and their lived experience. This may be relevant given that the participants’ tolerance to uncertainty is unknown but may influence their pain intensity and disability [81]. Most participants were willing to change behaviours to improve their pain. Evidence suggests however that while behavioural change interventions may be effective in improving exercise adherence, [82, 83] people living with persistent pain understandably find behavioural change difficult [84]. Interventions involving behavioural change techniques have limited effects on pain and disability [85, 86], which may need attention to ensure approaches are person-centred and meaningful to the individual. Interestingly, a similar proportion of participants believed manual therapy and exercise may reduce future pain, however, there is limited evidence for manual therapy [40] and stronger evidence supporting exercise for prevention [36, 39].

Activity avoidance was perceived as important for reduction of future pain by over three-quarters of participants. Whether this reflects an appropriate response in terms of adjusting loading to care for pain (e.g. managing optimal load in an arthritic joint), or fear-avoidance beliefs in unclear, as this may relate to specific individual’s experience and perceptions. Fear-avoidance beliefs were potentially evident in other answers (e.g. “*You should be very careful exercising when you have pain*” (90% agreement) – see Additional file) but may reflect adaptive responses in some i.e., appropriate adjustment to loading in someone with joint pain or tendinopathy. The fear-avoidance model associates such beliefs with pain catastrophising [87], although it may not be the only pathway. It is interesting, therefore, that in this cohort the median PCS score was 16-points, which is not considered clinically-relevant [34] and is similar to healthy controls [88]. The association between pain catastrophising and fear-avoidance beliefs has been challenged [87], possibly consistent with evidence suggesting fear-avoidance beliefs may relate to avoidance of pain exacerbation without concern regarding tissue damage, or reflect individuals’ uncertainty about how to care for their pain [89].

### Dominance of healthcare professionals for providing healthcare guidance

Participants felt it very important to seek advice and treatments from healthcare professionals for pain, with few endorsing self-care as their highest-ranking care strategy. Similarly, few participants gave high importance to the internet for guiding pain care and even fewer accessed or were aware of evidence-based websites. One quarter of participants did not consider scientific evidence at all in healthcare decision-making (see Additional file). These findings suggest the ideal modes of public health information delivery regarding pain needs further exploration to understand how to mobilise evidence that is meaningful and important to the community.

### Comparison of those with and without pain

Consistent with previous research, the participants with pain in the present study were significantly more likely to be older and have lower levels of education [13, 90, 91]. Those with no pain appeared more likely to perceive that most pain gets better, that beliefs about injury/tissue damage can influence pain, that psychological treatments can be helpful for pain care and that future pain episodes can be avoided. People with no pain showed significantly less pain catastrophising (particularly helplessness). These findings reflect a more optimistic, possibly protective [92], view of pain, which may have been influenced by their lived experience. However, those with no pain were less likely to “carry on doing what I’m doing no matter what” if they did get pain. We are unable to determine from the survey why people with no pain may change their activities should they develop pain.

### Strengths and limitations

This study took a novel approach to understanding pain by surveying the public across a broad range of musculoskeletal pain-related domains. Previous public surveys have focussed on specific pain disorders [26, 27]. The questionnaire utilised closed questions and differing information may have been collected had participants been able to give free text responses, or had semi-structured interviews been undertaken.

A high proportion of participants had musculoskeletal pain at the time of survey completion or reported pain of at least three-months duration in the previous year. These prevalence statistics are higher than reported elsewhere within Europe [93, 94], possibly due to persons living with or having lived with pain being more inclined to complete this survey. Pain interference was significant for nearly two-thirds of the sample, however, the majority had taken minimal time off work because of pain-related symptoms. Therefore, data for this cohort should be considered as predominantly consisting of community-dwelling people with musculoskeletal pain. Recent / current care-seeking was not captured in this questionnaire.

Apparent themes should be considered in the context of where the research was undertaken and sample characteristics. As a small, contained, Westernised community Guernsey was considered an easy location to recruit a high proportion of the public through multimedia advertising (for example approximately 75% of the population access the island’s newspaper [95]). Compared to previous pain-related public surveys undertaken [26, 27] a large proportion (up to 2.71%) of Guernsey’s public participated. Guernsey is autonomous, but a dependency of the British Crown. It is not in the European Union. The States of Guernsey are the island’s executive authority responsible for legislature and include a Committee for Health and Social Care. Access to primary healthcare (general practitioners, physiotherapists etc.) is not government-funded, while secondary healthcare (hospitalisation, consultant-level care etc.) is government-funded and free at point-of-use. To facilitate comparison with other jurisdictions further Guernsey sociodemographic data are outlined in the Additional file. Participants’ age range was similar to the island’s population, however, the sample contained approximately 76% female participants which is not representative [96]. A large proportion (65.4%) of participants had persistent pain, possibly reflecting participation bias. While the prevalence of pain disorders is higher in females this is usually in the region of 5.5% more than males [61]. In Europe, it is estimated that 56% of people with chronic pain are female [93]. Social media advertising contributed greatly to participant recruitment, however, it is estimated that approximately 56% of Facebook users are male [97]. We are therefore unable to explain the high female participation. Ethnicity data were not collected in this questionnaire, however, population level ‘place of birth’ data is available (see Additional file).

Given that the determination of whether participants views were aligned with the contemporary literature, or not, was based upon the researchers’ interpretation of the literature, a position statement for the researchers is given in the Additional file.

## Conclusions

Implications of this research can be considered at two levels. This study suggests clinicians should ascertain patients’ beliefs particularly regarding prognosis. Also, even if they appear to have some biomedical beliefs, our findings suggest that patients may be open to considering broader multidimensional influences on their pain. Patients are likely to be open to therapeutic exercise, but need guidance about what is appropriate/safe. Clinicians should not assume patients want certain treatments (e.g. manual therapy) and illuminate the need to utilise a shared decision-making process taking into account an individual’s beliefs, preferences and expectations to deliver targeted (self)-management that addresses their specific needs and priorities.

Public health campaigns may help to facilitate a broader community-wide contemporary multidimensional understanding of pain. The public’s knowledge of pain self-care could be facilitated together with greater knowledge regarding realistic prognoses/recurrences and evidence-based reduction of recurrences. A good example here is the global Choosing Wisely initiative (e.g. https://www.choosingwisely.org.au/) that aims to support people to choose evidence-based, high-value healthcare and reduce unnecessary or ineffective tests and treatments. The study highlights a need for improvements into how we understand complex belief patterns, the public’s multidimensional understanding of pain and the individualisation of pain care. The public also needs to be able to access reliable, credible and trustworthy evidence-based pain information through means other than their healthcare practitioners.

### Supplementary Information

**Additional file 1.** Rabey et al. 2024 – Guernsey Pain Survey.

## Data Availability

The datasets generated and/or analysed during the current study are not publicly available due to an absence of ethics committee clearance for public deposition but are available from the corresponding author on reasonable request.

## Abbreviations

A: Agree
BPS: Biopsychosocial
D: Disagree]
IQR: Inter-quartile range
Max: Maximum
Med: Biomedical
Min: Minimum
N: Neither agree nor disagree / neutral
PCS: Pain Catastrophising Scale
SMA: Somewhat agree
SMD: Somewhat disagree
STA: Strongly agree
STD: Strongly disagree
