# Supplementary material for: Societal beliefs about pain may be more balanced than previously thought. Results of the Guernsey pain survey
Source: BMC Musculoskelet Disord. 2024 Jan 18;25:72. doi: 10.1186/s12891-023-07088-0 (PMC10795459; doi:10.1186/s12891-023-07088-0)
Supplement: Supplementary file 1 — Additional file 1. Rabey et al. 2024 – Guernsey Pain Survey. [file 12891_2023_7088_MOESM1_ESM.pdf]

Additional Files– Rabey et al. 2024 – Guernsey Pain Survey

Contents

Questionnaire 2

Demographics 2

Pain experience 2

Understanding of pain 2

Understanding of pain treatment 4

Influence on pain 6

Physical activity and pain 7

Pain Catastrophising Scale (PCS) 8

Healthcare decision making 8

References 10

Research teams' answers to questionnaire, comments and bibliography. 11

Understanding of pain. 12

Understanding of pain treatments. 15

Beliefs about factors influencing pain 19

References 21

Full results tables 29

Respondents' understanding of pain 29

Respondents' understanding of pain treatments. 32

Respondents' beliefs about factors influencing pain 36

Respondents' beliefs about physical activity and pain 38

Results from the Pain Catastrophising Scale including comparisons between the pain subgroup and the no pain subgroup 39

Factors influencing healthcare decision making 42

Guernsey sociodemographic data (2019-2020) to facilitate comparison with other jurisdictions. .. 45

Age and sex distribution of Guernsey population at March 2019[1] 47

References: 48

Authors' position statement 49

Questionnaire

Demographics

- Age.
- Gender.
- Highest educational level attained (primary school, high school, trade / vocational qualification, university degree, postgraduate degree).
- Participants were asked whether they were an exercise prescriber (e.g. personal trainer), complementary / alternative therapist or registered healthcare professional.

Pain experience

- Participants were asked whether they currently had any musculoskeletal pain or had ever experienced pain lasting three months or more. They were also asked whether they had had pain lasting three months or more during the past year, and if so how much this pain had interfered with daily activities (A lot, Quite a bit, A little, Not at all).
- Taken from the Orebro Musculoskeletal Pain Screening Questionnaire [53] participants were asked, "How many days of work have you missed because of pain in the last 12 months?"

Understanding of pain

Participants indicated their beliefs (true, false, unsure) regarding several statements.

The following statements were taken from the Revised Neurophysiology of Pain Questionnaire [16]:

- Pain ***only*** occurs when you are injured or at risk of being injured.

- Persistent pain means that an injury hasn't healed properly.
- The body tells the brain when it is in pain.
- Pain occurs whenever you are injured.
- When you injure yourself, the environment that you are in will not affect the amount of pain you experience, as long as the injury is exactly the same.
- The brain decides when you will experience pain.

The following two statements were taken from the Avoidance-Endurance Questionnaire [35]:

- When I have pain I think to myself "don't make such a fuss"
- When I have pain I carry on doing what I'm doing no matter what

The following statements were developed to further explore public beliefs about pain:

- There is always a simple explanation for why someone has pain
- Most pain gets better
- Once you have pain, you're always likely to have pain
- More pain means more tissue damage (i.e. damage to joints, nerves, tendons or muscles)
- There is always tissue damage to explain pain
- It is possible to have tissue damage but no pain
- Findings on scans like arthritis and disc bulges are always associated with pain
- An increase in pain is an indication that you should stop doing what you're doing until the pain decreases

- Pain may mean something is out of place
- If pain is not associated with injury or tissue damage it must be psychological
- Pain is abnormal
- Pain means you aren't healthy
- Pain is usually caused by physical overuse or excessive strain
- Pain is usually caused by work or by an accident at work
- Full pain relief is necessary before returning to work, sport or other daily activities
- Tests like MRI scans, x-rays and ultrasound imaging are critical to identify the source of pain
- The source of pain must always be identified for adequate pain treatment to occur
- It is possible to have the right treatment for pain without having tests like MRI scans, x-rays or ultrasound imaging
- When I am in pain my family/partner should look after me

Understanding of pain treatment

Participants indicated their beliefs (true, false, unsure) regarding the following statements developed to explore public beliefs about pain treatment:

- It is important to rest when you have pain
- It is important to stay active when you have pain
- It is important to gradually increase your activity when you have pain
- If you experience pain, you should just keep pushing through
- It is possible to manage pain well yourself

- It is important to seek professional advice for pain care
- It is important to seek treatments (medications, injections, surgery, hands-on treatments) from professionals to get pain relief

The following three statements were replicated for several interventions (medication, injections, surgery, exercise, psychological treatments, “hands-on” therapies):

- Medications can be helpful for treating pain
- Medications are always helpful for treating pain
- Medications are never necessary for treating pain
- Stronger medications are always better for pain relief
- Surgery should only be considered as a final option when other treatments have not worked
- There is always some surgical procedure or medication that will get rid of pain
- You should be very careful exercising when you have pain
- Stretching is always an effective exercise for pain
- Good core stability is key to managing pain
- It is always important to maintain good alignment when exercising, especially if you have pain
- Good advice can be sufficient pain care
- Understanding how pain works is an effective pain treatment
- Relaxation and mental distraction are good ways of treating pain
- Psychological treatments (talk therapies, stress management, mindfulness) should only be used for pain relief when nothing else has worked

- Addressing mood and stress/anxiety is important for good pain care
- Physical therapies (physiotherapy, osteopathy, chiropractic) should always include 'hands-on' treatments for pain relief
- It is important to treat underlying lifestyle factors for pain relief (e.g. sleep, stress, work habits, exercise, diet)
- I am usually willing to change my habits and behaviours to improve my health and pain care
- Future episodes of pain can be reduced or avoided by avoiding aggravating activities
- Future episodes of pain can be reduced or avoided through exercise
- Future episodes of pain can be reduced or avoided by getting regular 'hands-on' treatments like massage or manipulation
- Future episodes of pain can be reduced or avoided by addressing lifestyle factors like sleep, weight and stress
- Future episodes of pain cannot be avoided

Influence on pain

Participants were asked to indicate whether they believe the following factors (which in the literature are associated with musculoskeletal pain) influence pain.:

- Mood
- Beliefs about injury and tissue damage
- Physical activity levels
- Posture and alignment (e.g. spinal posture, leg alignment, foot posture)
- Sleep

- Diet
- Weak core muscles
- Muscle tightness
- Age
- Social support
- Beliefs about pain
- Weight
- Stress (at home, at work etc)
- Access to appropriate healthcare
- Ergonomics (e.g. work set up and practices)
- Culture
- Amount of tissue damage or injury (e.g. disc bulges, arthritis, tendon strains etc.)
- Whether you are male of female
- Education level
- Other health problems (e.g. heart disease, diabetes, lung conditions etc)
- Alcohol/drug consumption
- Genetics

Physical activity and pain

Three questions were taken from the Fear-Avoidance Beliefs Questionnaire [91]. Participants were asked to indicate their level of agreement with the following statements, using a seven=point scale ranging from, "Strongly disagree," to, "Strongly agree."

- Physical activity makes pain worse.
- Physical activity might harm my body if I am in pain.
- I should not do physical activities which (might) make pain worse.

Pain Catastrophising Scale (PCS)

Pain catastrophising was assessed using the full Pain Catastrophising scale (PCS), a valid and reliable [84] questionnaire examining a person's thoughts and feelings in terms of magnification, rumination, and helplessness about pain. On a 0 - 4 scale participants indicate the frequency at which they experience these different types of catastrophic thoughts described in 13 statements, giving a total score of 0 - 52 points, with higher scores reflecting greater pain catastrophising.

Healthcare decision making

Participants were asked, "What influences your decisions to have certain types of treatments?" Participants ranked the following statements in order of importance with "1" being the most important. They were able to indicate that an answer was not applicable to them.

- Recommendations from friends / family
- Recommendations from my doctor
- Recommendations from my other registered health professional (nurse, physiotherapist, osteopath, chiropractor etc.)
- Recommendations from my alternative/complementary therapist

- General internet search
- I look for treatments that have a lot of scientific evidence
- I research treatments on websites like Cochrane, Pedro, NHS Choices, NICE guidelines
- I look for treatments that seem easy and are likely to have fast effects
- I look for treatments that are least invasive
- I look for treatments where I can take control and learn how to self-manage

References

[1] Catley MJ, O'Connell NE, Moseley GL. How good is the neurophysiology of pain questionnaire? A Rasch analysis of psychometric properties. J Pain 2013;14(8):818-827.

[2] Hasenbring MI, Hallner D, Rusu AC. Fear-avoidance- and endurance-related responses to pain: Development and validation of the Avoidance-Endurance Questionnaire (AEQ). European Journal of Pain 2009;13:620-628.

[3] Linton S, Boersma K. Early Identification of Patients at Risk of Developing a Persistent Back Problem: The Predictive Validity of The Örebro Musculoskeletal Pain Questionnaire. Clinical Journal of Pain 2003;19:80-86.

[4] Sullivan M, Bishop S, Pivik J. The Pain Catastrophizing Scale: Development and Validation. Psychological Assessment 1995;7(4):524-532.

[5] Waddell G, Newton M, Henderson I, Somerville D, Main CJ. A Fear-Avoidance Beliefs Questionnaire (FABQ) and the role of fear-avoidance beliefs in chronic low back pain and disability. PAIN 1993;52(2):157-168.

Research teams’ answers to questionnaire, comments and bibliography.

For each statement in the survey, based upon review of contemporary literature, the research team members were tasked with determining whether each item was true / false (reflecting a contemporary multidimensional view of pain and pain management)/ or equivocal (possibly reflecting conflicting decisions based on context). Members were also tasked with indicating whether the item reflected a dominantly biopsychosocial /biomedical / neutral or neither perspective. Members’ comments are shown where relevant, and references reflecting evidence relevant to those answers, are provided where applicable.

Understanding of pain.

Statement	True / False / Equivocal	Biopsychosocial (BPS) / Biomedical (Med) / Neutral or neither (N)	References	Comments
Pain only occurs when you are injured or at risk of being injured	False	Med	[25]	
Persistent pain means that an injury hasn't healed properly	Equivocal	Med		May be true, e.g. non-union of fracture, post-surgical complication etc.
Pain always occurs when you are injured	False	Med		
When you injure yourself, the environment that you are in will not affect the amount of pain you experience, as long as the injury is exactly the same	False	Med		
The brain decides when you will experience pain	True	BPS		
There is always a simple explanation for why someone has pain	False	Med	[80]	
Most pain gets better	True	N	[50]	
Once you have pain, you're always likely to have pain	False	N	[50; 63]	
More pain means more tissue damage (i.e., damage to joints, nerves, tendons or muscles)	False	Med	[14; 49]	

There is always tissue damage to explain pain	False	Med		
It is possible to have tissue damage but no pain	True	BPS	[14]	
Findings on scans like arthritis and disc bulges are always associated with pain	False	Med	[14]	
An increase in pain is an indication that you should stop doing what you're doing until the pain decreases	Equivocal	Med		May be interpreted differently – e.g. true for acute pain following tissue trauma, or inflammatory conditions (eg JIA) whilst false for persistent pain
Pain may mean something is out of place	False	Med	[90]	
If pain is not associated with injury or tissue damage it must be psychological	False	Med	[40; 72]	
Pain is abnormal	False	Med	[7]	
Pain means you aren't healthy	False	Med	[7]	
Pain is usually caused by physical overuse or excessive strain	False	Med		
Pain is usually caused by work or by an accident at work	False	Med		
Full pain relief is necessary before returning to work, sport or other daily activities	False	Med	[4]	
Tests like MRI scans, x-rays and ultrasound imaging are critical to identify the source of pain	False	Med	[14; 49]	

The source of pain must always be identified for adequate pain treatment to occur	False	Med		
It is possible to have the right treatment for pain without having tests like MRI scans, x-rays or ultrasound imaging	True	BPS	[32]	
When I have pain I think to myself “don’t make such a fuss”	False	N	[70]	
When I have pain I carry on doing what I’m doing no matter what	False	N	[70]	
When I am in pain my family/partner should look after me	False	N	[71]	

Understanding of pain treatments.

Statement	True / False / Equivocal	Biopsychosocial (BPS) / Biomedical (Med) / Neutral or neither (N)	References	Comments
It is important to rest when you have pain	Equivocal	Med		May be interpreted differently – e.g. true for acute pain following tissue trauma, inflammatory conditions; post-operative pain; whilst false for persistent pain
It is important to stay active when you have pain	True	BPS	[20]	
It is important to gradually increase your activity when you have pain	True	N	[54]	
If you experience pain, you should just keep pushing through	False	N		
It is possible to manage pain well yourself	True	N	[64]	
It is important to seek professional advice for pain care	Equivocal	N		May be credible digital information accessible if appropriate and no red flags.
It is important to seek treatments (medications, injections, surgery, hands-on treatments) from professionals to get pain relief	False	N		This may be context specific - e.g. cauda equina syndrome
Medications can be helpful for treating pain	True	Med	[6; 17; 31; 69]	
Medications are always helpful for treating pain	False	Med	[1; 2; 17; 27; 69]	

Medications are never necessary for treating pain	False	N	[76]	
Stronger medications are always better for pain relief	False	Med	[17; 69]	
Injections can be helpful for treating pain	True	Med	[6; 30; 46; 66]	
Injections are always helpful for treating pain	False	Med	[6; 30; 46; 66]	
Injections are never necessary for treating pain	False	N		
Surgery can be helpful for treating pain	True	Med	[26; 37]	
Surgery is always helpful for treating pain	False	Med	[26]	
Surgery is never necessary for treating pain	False	N	[37]	
Surgery should only be considered as a final option when other treatments have not worked	False	Med	[37]	
There is always some surgical procedure or medication that will get rid of pain	False	Med	[1; 2; 17; 26]	
Exercise can be helpful for treating pain	True	N	[31; 36]	
Exercise is always helpful for treating pain	False	N	[52]	
Exercise is never necessary for treating pain	False	N		
You should be very careful exercising when you have pain	Equivocal	Med		May be interpreted differently – e.g. true for acute pain following tissue trauma, whilst false for persistent pain.
Stretching is always an effective exercise for pain	False	Med	[56; 67]	
Good core stability is key to managing pain	False	Med	[75]	
It is always important to maintain good alignment when exercising, especially if you have pain	False	Med	[39; 74]	

Good advice can be sufficient pain care	True	N	[51]
Understanding how pain works is an effective pain treatment	Equivocal	N	[22; 45; 86; 92]
Relaxation and mental distraction are good ways of treating pain	True	BPS	[87]
Psychological treatments (talk therapies, stress management, mindfulness) can be helpful for treating pain	True	BPS	[94]
Psychological treatments are always helpful for treating pain	False	BPS	
Psychological treatments are never necessary for treating pain	False	Med	
Psychological treatments should only be used for pain relief when nothing else has worked	False	Med	
Addressing mood and stress/anxiety is important for good pain care	True	BPS	[48]
‘Hands-on' therapies (massage, manipulation) can be helpful for treating pain	True	Med	[31; 73]
‘Hands-on' therapies are always helpful for treating pain	False	Med	
‘Hands-on' therapies are never necessary for treating pain	False	N	[31; 73]
Physical therapies (physiotherapy, osteopathy, chiropractic) should always include ‘hands-on’ treatments for pain relief	False	Med	[31; 73]
It is important to treat underlying lifestyle factors for pain relief (e.g. sleep, stress, work habits, exercise, diet)	True	BPS	[72; 89]
I am usually willing to change my habits and behaviours to improve my health and pain care	Equivocal	BPS	[3; 59]

Future episodes of pain can be reduced or avoided by avoiding aggravating activities	False	Med		
Future episodes of pain can be reduced or avoided through exercise	True	N	[78; 82]	
Future episodes of pain can be reduced or avoided by getting regular 'hands-on' treatments like massage or manipulation	False	Med	[41]	
Future episodes of pain can be reduced or avoided by addressing lifestyle factors like sleep, weight and stress	Equivocal	BPS	[29]	
Future episodes of pain cannot be avoided	True	N	[8; 9; 19; 44]	Recurrence of symptoms likely.

Beliefs about factors influencing pain

Influencing factor?	True / False / Equivocal	Biopsychosocial (BPS) / Biomedical (Med) / Neutral or neither (N)	References	Comments
Mood	True	BPS	[5; 15; 24; 68; 83]	
Beliefs about injury and tissue damage	True	BPS		
Posture and alignment (e.g. spinal posture, leg alignment, foot posture)	Equivocal	Med	[57; 62]	
Sleep	True	BPS	[88]	
Diet	True	N	[12]	
Weak muscles	True	Med	[23; 38; 55; 65]	
Muscle tightness	False	Med	[10; 81]	
Age	True	Med	[43]	
Social support (support / lack of support from family, friends, colleagues, others)	True	BPS	[71]	
How you think about pain	True	BPS	[33; 42; 58; 93]	
Weight	True	Med	[79; 96]	
Stress (at home, at work etc)	True	BPS	[11]	
Access to appropriate healthcare	True	N	[13]	
Ergonomics (e.g. work set up and practices)	Equivocal	Med	[68]	

Culture (society's beliefs and practices)	True	BPS	[77]
Amount of tissue damage or injury (e.g., disc bulges, arthritis, tendon, muscle, ligament strains)	True	Med	[34; 49]
Whether you are male or female	True	Med	[28; 61]
Education level	True	BPS	[18]
Other health problems (e.g. heart disease, diabetes, lung conditions etc)	True	BPS / Med	[85]
Alcohol or drug use	True	Med	[21; 47; 95]
Genetics	True	Med	[60]
None of the above	False	n/a	

References

[1] Abdel Shaheed C, Awal W, Zhang G, Gilbert SE, Gallacher D, McLachlan A, Day RO, Ferreira GE, Jones CM, Ahedi H, Tamrakar M, Blyth FM, Stanaway F, Maher CG. Efficacy, safety, and dose-dependence of the analgesic effects of opioid therapy for people with osteoarthritis: systematic review and meta-analysis. Medical Journal of Australia 2022;216(6):305-311.

[2] Abdel Shaheed C, Ferreira GE, Dmitritchenko A, McLachlan AJ, Day RO, Saragiotto B, Lin C, Langendyk V, Stanaway F, Latimer J, Kamper S, McLachlan H, Ahedi H, Maher CG. The efficacy and safety of paracetamol for pain relief: an overview of systematic reviews. Med J Aust 2021;214(7):324-331.

[3] Andrews NE, Strong J, Meredith PJ, Gordon K, Bagraith KS. "It's very hard to change yourself": an exploration of overactivity in people with chronic pain using interpretative phenomenological analysis. Pain 2015;156(7):1215-1231.

[4] Ardern CL, Glasgow P, Schneiders A, Witvrouw E, Clarsen B, Cools A, Gojanovic B, Griffin S, Khan KM, Moksnes H, Mutch SA, Phillips N, Reurink G, Sadler R, Grävare Silbernagel K, Thorborg K, Wangensteen A, Wilk KE, Bizzini M. 2016 Consensus statement on return to sport from the First World Congress in Sports Physical Therapy, Bern. British Journal of Sports Medicine 2016;50(14):853-864.

[5] Atherton K, Wiles NJ, Lecky FE, Hawes SJ, Silman AJ, Macfarlane GJ, Jones GT. Predictors of persistent neck pain after whiplash injury. Emerg Med J 2006;23(3):195-201.

[6] Babatunde OO, Jordan JL, Van der Windt DA, Hill JC, Foster NE, Protheroe J. Effective treatment options for musculoskeletal pain in primary care: A systematic overview of current evidence. PloS one 2017;12(6):e0178621-e0178621.

[7] Baldwin JN, McKay MJ, Burns J, Hiller CE, Nightingale EJ, Moloney N. What are the similarities and differences between healthy people with and without pain? Scand J Pain 2018;18(1):39-47.

[8] Bastick AN, Verkleij SP, Damen J, Wesseling J, Hilberdink WK, Bindels PJ, Bierma-Zeinstra SM. Defining hip pain trajectories in early symptomatic hip osteoarthritis--5 year results from a nationwide prospective cohort study (CHECK). Osteoarthritis Cartilage 2016;24(5):768-775.

[9] Bastick AN, Wesseling J, Damen J, Verkleij SP, Emans PJ, Bindels PJ, Bierma-Zeinstra SM. Defining knee pain trajectories in early symptomatic knee osteoarthritis in primary care: 5-year results from a nationwide prospective cohort study (CHECK). Br J Gen Pract 2016;66(642):e32-39.

[10] Bhimani R, Gaugler JE, Felts J. Consensus Definition of Muscle Tightness From Multidisciplinary Perspectives. Nursing Research 2020;69(2).

[11] Boakye PA, Olechowski C, Rashiq S, Verrier MJ, Kerr B, Witmans M, Baker G, Joyce A, Dick BD. A Critical Review of Neurobiological Factors Involved in the Interactions Between Chronic Pain, Depression, and Sleep Disruption. Clin J Pain 2016;32(4):327-336.

[12] Brain K, Burrows TL, Rollo ME, Chai LK, Clarke ED, Hayes C, Hodson FJ, Collins CE. A systematic review and meta-analysis of nutrition interventions for chronic noncancer pain. J Hum Nutr Diet 2019;32(2):198-225.

[13] Briggs AM, Slater H, Hsieh E, Kopansky-Giles D, Åkesson KE, Dreinhöfer KE, March LM, Woolf AD. System strengthening to support value-based care and healthy ageing for people with chronic pain. Pain 2019;160(6):1240-1244.

[14] Brinjikji W, Luetmer PH, Comstock B, Bresnahan BW, Chen LE, Deyo RA, Halabi S, Turner JA, Avins AL, James K, Wald JT, Kallmes DF, Jarvik JG. Systematic literature review of imaging features of spinal degeneration in asymptomatic populations. AJNR Am J Neuroradiol 2015;36(4):811-816.

[15] Campbell P, Bishop A, Dunn KM, Main CJ, Thomas E, Foster NE. Conceptual overlap of psychological constructs in low back pain. PAIN 2013;154(9):1783-1791.

[16] Catley MJ, O'Connell NE, Moseley GL. How good is the neurophysiology of pain questionnaire? A Rasch analysis of psychometric properties. J Pain 2013;14(8):818-827.

[17] Chou R, Deyo R, Friedly J, Skelly A, Weimer M, Fu R, Dana T, Kraegel P, Griffin J, Grusing S. Systemic Pharmacologic Therapies for Low Back Pain: A Systematic Review for an American College of Physicians Clinical Practice Guideline. Ann Intern Med 2017;166(7):480-492.
[18] Costa L, Henschke N, Maher C, Refshauge K, Herbert R, McAuley J, Das A, Costa L. Prognosis of chronic low back pain: design of an inception cohort study. BMC Musculoskeletal Disorders 2007;8(1):11.
[19] da Silva T, Mills K, Brown BT, Herbert RD, Maher CG, Hancock MJ. Risk of Recurrence of Low Back Pain: A Systematic Review. J Orthop Sports Phys Ther 2017;47(5):305-313.
[20] Dahm KT, Brurberg KG, Jamtvedt G, Hagen KB. Advice to rest in bed versus advice to stay active for acute low-back pain and sciatica. Cochrane Database of Systematic Reviews 2010(6).
[21] Dassieu L, Kaboré J-L, Choinière M, Arruda N, Roy É. Understanding the link between substance use and chronic pain: A qualitative study among people who use illicit drugs in Montreal, Canada. Drug and Alcohol Dependence 2019;202:50-55.
[22] de Oliveira Lima L, Saragiotto BT, Costa LOP, Nogueira LC, Meziat-Filho N, Reis FJJ. Self-Guided Web-Based Pain Education for People With Musculoskeletal Pain: A Systematic Review and Meta-Analysis. Phys Ther 2021;101(10).
[23] de Sousa CS, de Jesus FLA, Machado MB, Ferreira G, Ayres IGT, de Aquino LM, Fukuda TY, Gomes-Neto M. Lower limb muscle strength in patients with low back pain: a systematic review and meta-analysis. J Musculoskelet Neuronal Interact 2019;19(1):69-78.
[24] Dell'Isola A, Steultjens M. Classification of patients with knee osteoarthritis in clinical phenotypes: Data from the osteoarthritis initiative. PLoS One 2018;13(1):e0191045.
[25] Elman I, Borsook D. Threat Response System: Parallel Brain Processes in Pain vis-à-vis Fear and Anxiety. Frontiers in Psychiatry 2018;9.
[26] Evans L, O'Donohoe T, Morokoff A, Drummond K. The role of spinal surgery in the treatment of low back pain. Med J Aust 2023;218(1):40-45.
[27] Ferreira GE, McLachlan AJ, Lin C-WC, Zadro JR, Abdel-Shaheed C, O'Keeffe M, Maher CG. Efficacy and safety of antidepressants for the treatment of back pain and osteoarthritis: systematic review and meta-analysis. BMJ 2021;372:m4825.
[28] Fillingim RB, King CD, Ribeiro-Dasilva MC, Rahim-Williams B, Riley JL. Sex, Gender, and Pain: A Review of Recent Clinical and Experimental Findings. The Journal of Pain 2009;10(5):447-485.
[29] Gatchel RJ, Neblett R, Kishino N, Ray CT. Fear-Avoidance Beliefs and Chronic Pain. Journal of Orthopaedic & Sports Physical Therapy 2016;46(2):38-43.
[30] Gaujoux-Viala C, Dougados M, Gossec L. Efficacy and safety of steroid injections for shoulder and elbow tendonitis: a meta-analysis of randomised controlled trials. Annals of the Rheumatic Diseases 2009;68(12):1843-1849.
[31] Gianola S, Bargeri S, Del Castillo G, Corbetta D, Turolla A, Andreano A, Moja L, Castellini G. Effectiveness of treatments for acute and subacute mechanical non-specific low back pain: a systematic review with network meta-analysis. British Journal of Sports Medicine 2022;56(1):41-50.
[32] Hall AM, Aubrey-Bassler K, Thorne B, Maher CG. Do not routinely offer imaging for uncomplicated low back pain. BMJ 2021;372:n291.
[33] Hallegraeff JM, Krijnen WP, van der Schans CP, de Greef MH. Expectations about recovery from acute non-specific low back pain predict absence from usual work due to chronic low back pain: a systematic review. J Physiother 2012;58(3):165-172.
[34] Hancock M, Maher C, Macaskill P, Latimer J, Kos W, Pik J. MRI findings are more common in selected patients with acute low back pain than controls? Eur Spine J 2012;21(2):240-246.
[35] Hasenbring MI, Hallner D, Rusu AC. Fear-avoidance- and endurance-related responses to pain: Development and validation of the Avoidance-Endurance Questionnaire (AEQ). European Journal of Pain 2009;13:620-628.

[36] Hayden J, Ellis J, Ogilvie R, Malmivaara A, van Tulder M. Exercise therapy for chronic low back pain. Cochrane Database of Systematic Reviews 2021;9:CD009790.
[37] Heath EL, Ackerman IN, Cashman K, Lorimer M, Graves SE, Harris IA. Patient-reported outcomes after hip and knee arthroplasty. Bone & Joint Open 2021;2(6):422-432.
[38] Hodges PW, Danneels L. Changes in Structure and Function of the Back Muscles in Low Back Pain: Different Time Points, Observations, and Mechanisms. Journal of Orthopaedic & Sports Physical Therapy 2019;49(6):464-476.
[39] Howe L, Lehman G. Getting out of neutral: the risks and rewards of lumbar spine flexion during lifting exercises. Strength and Conditioning. Strength and Conditioning 2021;March.
[40] IASP Taxonomy Working Group. IASP Taxonomy, 2011.
[41] Iben A, Lise H, Charlotte L-Y. Chiropractic maintenance care - what's new? A systematic review of the literature. Chiropractic & manual therapies 2019;27:63-63.
[42] Jackson T, Wang Y, Wang Y, Fan H. Self-efficacy and chronic pain outcomes: a meta-analytic review. J Pain 2014;15(8):800-814.
[43] James SL, Abate D, Abate KH, Abay SM, Abbafati C, Abbasi N, Abbastabar H, Abd-Allah F, Abdela J, Abdelalim A, Abdollahpour I, Abdulkader RS, Abebe Z, Abera SF, Abil OZ, Abraha HN, Abu-Raddad LJ, Abu-Rmeileh NME, Accrombessi MMK, Acharya D, Acharya P, Ackerman IN, Adamu AA, Adebayo OM, Adekanmbi V, Adetokunboh OO, Adib MG, Adsuar JC, Afanvi KA, Afarideh M, Afshin A, Agarwal G, Agesa KM, Aggarwal R, Aghayan SA, Agrawal S, Ahmadi A, Ahmadi M, Ahmadieh H, Ahmed MB, Aichour AN, Aichour I, Aichour MTE, Akinyemiju T, Akseer N, Al-Aly Z, Al-Eyadhy A, Al-Mekhlafi HM, Al-Raddadi RM, Alahdab F, Alam K, Alam T, Alashi A, Alavian SM, Alene KA, Alijanzadeh M, Alizadeh-Navaei R, Aljunid SM, Alkerwi Aa, Alla F, Allebeck P, Alouani MML, Altirkawi K, Alvis-Guzman N, Amare AT, Aminde LN, Ammar W, Amoako YA, Anber NH, Andrei CL, Androudi S, Animut MD, Anjomshoa M, Ansha MG, Antonio CAT, Anwari P, Arabloo J, Arauz A, Aremu O, Ariani F, Armoon B, Ärnlöv J, Arora A, Artaman A, Aryal KK, Asayesh H, Asghar RJ, Ataro Z, Atre SR, Ausloos M, Avila-Burgos L, Avokpaho EFGA, Awasthi A, Ayala Quintanilla BP, Ayer R, Azzopardi PS, Babazadeh A, Badali H, Badawi A, Bali AG, Ballesteros KE, Ballew SH, Banach M, Banoub JAM, Banstola A, Barac A, Barboza MA, Barker-Collo SL, Bärnighausen TW, Barrero LH, Baune BT, Bazargan-Hejazi S, Bedi N, Beghi E, Behzadifar M, Behzadifar M, Béjot Y, Belachew AB, Belay YA, Bell ML, Bello AK, Bensenor IM, Bernabe E, Bernstein RS, Beuran M, Beyranvand T, Bhala N, Bhattarai S, Bhaumik S, Bhutta ZA, Biadgo B, Bijani A, Bikbov B, Bilano V, Bililign N, Bin Sayeed MS, Bisanzio D, Blacker BF, Blyth FM, Bou-Orm IR, Boufous S, Bourne R, Brady OJ, Brainin M, Brant LC, Brazinova A, Breitborde NJK, Brenner H, Briant PS, Briggs AM, Briko AN, Britton G, Brugha T, Buchbinder R, Busse R, Butt ZA, Cahuana-Hurtado L, Cano J, Cárdenas R, Carrero JJ, Carter A, Carvalho F, Castañeda-Orjuela CA, Castillo Rivas J, Castro F, Catalá-López F, Cercy KM, Cerin E, Chaiah Y, Chang AR, Chang H-Y, Chang J-C, Charlson FJ, Chattopadhyay A, Chattu VK, Chaturvedi P, Chiang PP-C, Chin KL, Chitheer A, Choi J-YJ, Chowdhury R, Christensen H, Christopher DJ, Cicuttini FM, Ciobanu LG, Cirillo M, Claro RM, Collado-Mateo D, Cooper C, Coresh J, Cortesi PA, Cortinovis M, Costa M, Cousin E, Criqui MH, Cromwell EA, Cross M, Crump JA, Dadi AF, Dandona L, Dandona R, Dargan PI, Daryani A, Das Gupta R, Das Neves J, Dasa TT, Davey G, Davis AC, Davitoiu DV, De Courten B, De La Hoz FP, De Leo D, De Neve J-W, Degefa MG, Degenhardt L, Deiparine S, Dellavalle RP, Demoz GT, Deribe K, Dervenis N, Des Jarlais DC, Dessie GA, Dey S, Dharmaratne SD, Dinberu MT, Dirac MA, Djalalinia S, Doan L, Dokova K, Doku DT, Dorsey ER, Doyle KE, Driscoll TR, Dubey M, Dubljanin E, Duken EE, Duncan BB, Duraes AR, Ebrahimi H, Ebrahimpour S, Echko MM, Edvardsson D, Effiong A, Ehrlich JR, El Bcheraoui C, El Sayed Zaki M, El-Khatib Z, Elkout H, Elyazar IRF, Enayati A, Endries AY, Er B, Erskine HE, Eshrati B, Eskandarieh S, Esteghamati A, Esteghamati S, Fakhim H, Fallah Omrani V, Faramarzi M, Fareed M, Farhadi F, Farid TA, Farinha CSEs, Farioli A, Faro A, Farvid MS, Farzadfar F, Feigin VL, Fentahun N, Fereshtehnejad S-M, Fernandes E, Fernandes JC, Ferrari AJ, Feyissa GT, Filip I, Fischer F, Fitzmaurice C, Foigt

NA, Foreman KJ, Fox J, Frank TD, Fukumoto T, Fullman N, Fürst T, Furtado JM, Futran ND, Gall S, Ganji M, Gankpe FG, Garcia-Basteiro AL, Gardner WM, Gebre AK, Gebremedhin AT, Gebremichael TG, Gelano TF, Geleijnse JM, Genova-Maleras R, Geramo YCD, Gething PW, Gezae KE, Ghadiri K, Ghasemi Falavarjani K, Ghasemi-Kasman M, Ghimire M, Ghosh R, Ghoshal AG, Giampaoli S, Gill PS, Gill TK, Ginawi IA, Giussani G, Gnedovskaya EV, Goldberg EM, Goli S, Gómez-Dantés H, Gona PN, Gopalani SV, Gorman TM, Goulart AC, Goulart BNG, Grada A, Grams ME, Grosso G, Gugnani HC, Guo Y, Gupta PC, Gupta R, Gupta R, Gupta T, Gyawali B, Haagsma JA, Hachinski V, Hafezi-Nejad N, Haghparast Bidgoli H, Hagos TB, Hailu GB, Haj-Mirzaian A, Haj-Mirzaian A, Hamadeh RR, Hamidi S, Handal AJ, Hankey GJ, Hao Y, Harb HL, Harikrishnan S, Haro JM, Hasan M, Hassankhani H, Hassen HY, Havmoeller R, Hawley CN, Hay RJ, Hay SI, Hedayatizadeh-Omran A, Heibati B, Hendrie D, Henok A, Herteliu C, Heydarpour S, Hibstu DT, Hoang HT, Hoek HW, Hoffman HJ, Hole MK, Homaie Rad E, Hoogar P, Hosgood HD, Hosseini SM, Hosseinzadeh M, Hostiuc M, Hostiuc S, Hotez PJ, Hoy DG, Hsairi M, Htet AS, Hu G, Huang JJ, Huynh CK, Iburg KM, Ikeda CT, Ileanu B, Ilesanmi OS, Iqbal U, Irvani SSN, Irvine CMS, Islam SMS, Islami F, Jacobsen KH, Jahangiry L, Jahanmehr N, Jain SK, Jakovljevic M, Javanbakht M, Jayatilleke AU, Jeemon P, Jha RP, Jha V, Ji JS, Johnson CO, Jonas JB, Jozwiak JJ, Jungari SB, Jürisson M, Kabir Z, Kadel R, Kahsay A, Kalani R, Kanchan T, Karami M, Karami Matin B, Karch A, Karema C, Karimi N, Karimi SM, Kasaeian A, Kassa DH, Kassa GM, Kassa TD, Kassebaum NJ, Katikireddi SV, Kawakami N, Karyani AK, Keighobadi MM, Keiyoro PN, Kemmer L, Kemp GR, Kengne AP, Keren A, Khader YS, Khafaei B, Khafaie MA, Khajavi A, Khalil IA, Khan EA, Khan MS, Khan MA, Khang Y-H, Khazaei M, Khoja AT, Khosravi A, Khosravi MH, Kiadaliri AA, Kiirithio DN, Kim C-I, Kim D, Kim P, Kim Y-E, Kim YJ, Kimokoti RW, Kinfu Y, Kisa A, Kissimova-Skarbek K, Kivimäki M, Knudsen AKS, Kocarnik JM, Kochhar S, Kokubo Y, Kolola T, Kopec JA, Kosen S, Kotsakis GA, Koul PA, Koyanagi A, Kravchenko MA, Krishan K, Krohn KJ, Kuate Defo B, Kucuk Bicer B, Kumar GA, Kumar M, Kyu HH, Lad DP, Lad SD, Lafranconi A, Lalloo R, Lallukka T, Lami FH, Lansingh VC, Latifi A, Lau KM-M, Lazarus JV, Leasher JL, Ledesma JR, Lee PH, Leigh J, Leung J, Levi M, Lewycka S, Li S, Li Y, Liao Y, Liben ML, Lim L-L, Lim SS, Liu S, Lodha R, Looker KJ, Lopez AD, Lorkowski S, Lotufo PA, Low N, Lozano R, Lucas TCD, Lucchesi LR, Lunevicius R, Lyons RA, Ma S, Macarayan ERK, Mackay MT, Madotto F, Magdy Abd El Razek H, Magdy Abd El Razek M, Maghavani DP, Mahotra NB, Mai HT, Majdan M, Majdzadeh R, Majeed A, Malekzadeh R, Malta DC, Mamun AA, Manda A-L, Manguerra H, Manhertz T, Mansournia MA, Mantovani LG, Mapoma CC, Maravilla JC, Marcenes W, Marks A, Martins-Melo FR, Martopullo I, März W, Marzan MB, Mashamba-Thompson TP, Massenburg BB, Mathur MR, Matsushita K, Maulik PK, Mazidi M, McAlinden C, McGrath JJ, McKee M, Mehndiratta MM, Mehrotra R, Mehta KM, Mehta V, Mejia-Rodriguez F, Mekonen T, Melese A, Melku M, Meltzer M, Memiah PTN, Memish ZA, Mendoza W, Mengistu DT, Mengistu G, Mensah GA, Mereta ST, Meretoja A, Meretoja TJ, Mestrovic T, Mezerji NMG, Miazgowski B, Miazgowski T, Millear AI, Miller TR, Miltz B, Mini GK, Mirarefin M, Mirrakhimov EM, Misganaw AT, Mitchell PB, Mitiku H, Moazen B, Mohajer B, Mohammad KA, Mohammadifard N, Mohammadnia-Afrouzi M, Mohammed MA, Mohammed S, Mohebi F, Moitra M, Mokdad AH, Molokhia M, Monasta L, Moodley Y, Moosazadeh M, Moradi G, Moradi-Lakeh M, Moradinazar M, Moraga P, Morawska L, Moreno Velásquez I, Morgado-Da-Costa J, Morrison SD, Moschos MM, Mountjoy-Venning WC, Mousavi SM, Mruts KB, Muche AA, Muchie KF, Mueller UO, Muhammed OS, Mukhopadhyay S, Muller K, Mumford JE, Murhekar M, Musa J, Musa KI, Mustafa G, Nabhan AF, Nagata C, Naghavi M, Naheed A, Nahvijou A, Naik G, Naik N, Najafi F, Naldi L, Nam HS, Nangia V, Nansseu JR, Nascimento BR, Natarajan G, Neamati N, Negoi I, Negoi RI, Neupane S, Newton CRJ, Ngunjiri JW, Nguyen AQ, Nguyen HT, Nguyen HLT, Nguyen HT, Nguyen LH, Nguyen M, Nguyen NB, Nguyen SH, Nichols E, Ningrum DNA, Nixon MR, Nolutshungu N, Nomura S, Norheim OF, Noroozi M, Norrving B, Noubiap JJ, Nouri HR, Nourollahpour Shiadeh M, Nowroozi MR, Nsoesie EO, Nyasulu PS, Odell CM, Ofori-Asenso R, Ogbo FA, Oh I-

H, Oladimeji O, Olagunju AT, Olagunju TO, Olivares PR, Olsen HE, Olusanya BO, Ong KL, Ong SK, Oren E, Ortiz A, Ota E, Otstavnov SS, Øverland S, Owolabi MO, P A M, Pacella R, Pakpour AH, Pana A, Panda-Jonas S, Parisi A, Park E-K, Parry CDH, Patel S, Pati S, Patil ST, Patle A, Patton GC, Paturi VR, Paulson KR, Pearce N, Pereira DM, Perico N, Pesudovs K, Pham HQ, Phillips MR, Pigott DM, Pillay JD, Piradov MA, Pirsaheb M, Pishgar F, Plana-Ripoll O, Plass D, Polinder S, Popova S, Postma MJ, Pourshams A, Poustchi H, Prabhakaran D, Prakash S, Prakash V, Purcell CA, Purwar MB, Qorbani M, Quistberg DA, Radfar A, Rafay A, Rafiei A, Rahim F, Rahimi K, Rahimi-Movaghar A, Rahimi-Movaghar V, Rahman M, Rahman MHu, Rahman MA, Rahman SU, Rai RK, Rajati F, Ram U, Ranjan P, Ranta A, Rao PC, Rawaf DL, Rawaf S, Reddy KS, Reiner RC, Reinig N, Reitsma MB, Remuzzi G, Renzaho AMN, Resnikoff S, Rezaei S, Rezai MS, Ribeiro ALP, Roberts NLS, Robinson SR, Roever L, Ronfani L, Roshandel G, Rostami A, Roth GA, Roy A, Rubagotti E, Sachdev PS, Sadat N, Saddik B, Sadeghi E, Saeedi Moghaddam S, Safari H, Safari Y, Safari-Faramani R, Safdarian M, Safi S, Safiri S, Sagar R, Sahebkar A, Sahraian MA, Sajadi HS, Salam N, Salama JS, Salamati P, Saleem K, Saleem Z, Salimi Y, Salomon JA, Salvi SS, Salz I, Samy AM, Sanabria J, Sang Y, Santomauro DF, Santos IS, Santos JV, Santric Milicevic MM, Sao Jose BP, Sardana M, Sarker AR, Sarrafzadegan N, Sartorius B, Sarvi S, Sathian B, Satpathy M, Sawant AR, Sawhney M, Saxena S, Saylan M, Schaeffner E, Schmidt MI, Schneider IJC, Schöttker B, Schwebel DC, Schwendicke F, Scott JG, Sekerija M, Sepanlou SG, Serván-Mori E, Seyedmousavi S, Shabaninejad H, Shafieesabet A, Shahbazi M, Shaheen AA, Shaikh MA, Shams-Beyranvand M, Shamsi M, Shamsizadeh M, Sharafi H, Sharafi K, Sharif M, Sharif-Alhoseini M, Sharma M, Sharma R, She J, Sheikh A, Shi P, Shibuya K, Shigematsu M, Shiri R, Shirkoohi R, Shishani K, Shiue I, Shokraneh F, Shoman H, Shrime MG, Si S, Siabani S, Siddiqi TJ, Sigfusdottir ID, Sigurvinsdottir R, Silva JP, Silveira DGA, Singam NSV, Singh JA, Singh NP, Singh V, Sinha DN, Skiadaresi E, Slepak ELN, Sliwa K, Smith DL, Smith M, Soares Filho AM, Sobaih BH, Sobhani S, Sobngwi E, Soneji SS, Soofi M, Soosaraei M, Sorensen RJD, Soriano JB, Soyiri IN, Sposato LA, Sreeramareddy CT, Srinivasan V, Stanaway JD, Stein DJ, Steiner C, Steiner TJ, Stokes MA, Stovner LJ, Subart ML, Sudaryanto A, Sufiyan MaB, Sunguya BF, Sur PJ, Sutradhar I, Sykes BL, Sylte DO, Tabarés-Seisdedos R, Tadakamadla SK, Tadesse BT, Tandon N, Tassew SG, Tavakkoli M, Taveira N, Taylor HR, Tehrani-Banihashemi A, Tekalign TG, Tekelemedhin SW, Tekle MG, Temesgen H, Temsah M-H, Temsah O, Terkawi AS, Teweldemedhin M, Thankappan KR, Thomas N, Tilahun B, To QG, Tonelli M, Topor-Madry R, Topouzis F, Torre AE, Tortajada-Girbés M, Touvier M, Tovani-Palone MR, Towbin JA, Tran BX, Tran KB, Troeger CE, Truelsen TC, Tsilimbaris MK, Tsoi D, Tudor Car L, Tuzcu EM, Ukwaja KN, Ullah I, Undurraga EA, Unutzer J, Updike RL, Usman MS, Uthman OA, Vaduganathan M, Vaezi A, Valdez PR, Varughese S, Vasankari TJ, Venketasubramanian N, Villafaina S, Violante FS, Vladimirov SK, Vlassov V, Vollset SE, Vosoughi K, Vujcic IS, Wagnew FS, Waheed Y, Waller SG, Wang Y, Wang Y-P, Weiderpass E, Weintraub RG, Weiss DJ, Weldegebreal F, Weldegwergs KG, Werdecker A, West TE, Whiteford HA, Widecka J, Wijeratne T, Wilner LB, Wilson S, Winkler AS, Wiyeh AB, Wiysonge CS, Wolfe CDA, Woolf AD, Wu S, Wu Y-C, Wyper GMA, Xavier D, Xu G, Yadgir S, Yadollahpour A, Yahyazadeh Jabbari SH, Yamada T, Yan LL, Yano Y, Yaseri M, Yasin YJ, Yeshaneh A, Yimer EM, Yip P, Yisma E, Yonemoto N, Yoon S-J, Yotebieng M, Younis MZ, Yousefifard M, Yu C, Zadnik V, Zaidi Z, Zaman SB, Zamani M, Zare Z, Zeleke AJ, Zenebe ZM, Zhang K, Zhao Z, Zhou M, Zodpey S, Zucker I, Vos T, Murray CJL. Global, regional, and national incidence, prevalence, and years lived with disability for 354 diseases and injuries for 195 countries and territories, 1990–2017: a systematic analysis for the Global Burden of Disease Study 2017. The Lancet 2018;392(10159):1789-1858.

[44] Johansson MS, Jensen Stochkendahl M, Hartvigsen J, Boyle E, Cassidy JD. Incidence and prognosis of mid-back pain in the general population: A systematic review. Eur J Pain 2017;21(1):20-28.

[45] Jones CMP, Shaheed CA, Ferreira GE, Kharel P, Christine Lin C-W, Maher CG. Advice and education provide small short-term improvements in pain and disability in people with non-specific spinal pain: a systematic review. Journal of Physiotherapy 2021;67(4):263-270.
[46] Jüni P, Hari R, Rutjes AW, Fischer R, Silletta MG, Reichenbach S, da Costa BR. Intra-articular corticosteroid for knee osteoarthritis. Cochrane Database Syst Rev 2015;2015(10):Cd005328.
[47] Karimi R, Mallah N, Nedjat S, Beasley MJ, Takkouche B. Association between alcohol consumption and chronic pain: a systematic review and meta-analysis. British Journal of Anaesthesia.
[48] Khoo E-L, Small R, Cheng W, Hatchard T, Glynn B, Rice DB, Skidmore B, Kenny S, Hutton B, Poulin PA. Comparative evaluation of group-based mindfulness-based stress reduction and cognitive behavioural therapy for the treatment and management of chronic pain: A systematic review and network meta-analysis. Evidence Based Mental Health 2019;22(1):26-35.
[49] Kjaer P, Leboeuf-Yde C, Korsholm L, Sorensen J, Bendix T. Magnetic resonance imaging and low back pain in adults: a diagnostic imaging study of 40-year-old men and women. Spine 2005;30:1173 - 1180.
[50] Lee JY, Walton DM, Tremblay P, May C, Millard W, Elliott JM, MacDermid JC. Defining pain and interference recovery trajectories after acute non-catastrophic musculoskeletal trauma through growth mixture modeling. BMC Musculoskeletal Disorders 2020;21(1):615.
[51] Liddle SD, Gracey JH, Baxter GD. Advice for the management of low back pain: a systematic review of randomised controlled trials. Man Ther 2007;12(4):310-327.
[52] Lima LV, Abner TSS, Sluka KA. Does exercise increase or decrease pain? Central mechanisms underlying these two phenomena. The Journal of Physiology 2017;595(13):4141-4150.
[53] Linton S, Boersma K. Early Identification of Patients at Risk of Developing a Persistent Back Problem: The Predictive Validity of The Örebro Musculoskeletal Pain Questionnaire. Clinical Journal of Pain 2003;19:80-86.
[54] López-de-Uralde-Villanueva I, Muñoz-García D, Gil-Martínez A, Pardo-Montero J, Muñoz-Plata R, Angulo-Díaz-Parreño S, Gómez-Martínez M, La Touche R. A Systematic Review and Meta-Analysis on the Effectiveness of Graded Activity and Graded Exposure for Chronic Nonspecific Low Back Pain. Pain Med 2016;17(1):172-188.
[55] Loureiro A, Mills PM, Barrett RS. Muscle weakness in hip osteoarthritis: A systematic review. Arthritis Care & Research 2013;65(3):340-352.
[56] Luan L, El-Ansary D, Adams R, Wu S, Han J. Knee osteoarthritis pain and stretching exercises: a systematic review and meta-analysis. Physiotherapy 2022;114:16-29.
[57] Mahmoud NF, Hassan KA, Abdelmajeed SF, Moustafa IM, Silva AG. The Relationship Between Forward Head Posture and Neck Pain: a Systematic Review and Meta-Analysis. Curr Rev Musculoskelet Med 2019;12(4):562-577.
[58] Martinez-Calderon J, Jensen MP, Morales-Asencio JM, Luque-Suarez A. Pain Catastrophizing and Function In Individuals With Chronic Musculoskeletal Pain: A Systematic Review and Meta-Analysis. Clin J Pain 2019;35(3):279-293.
[59] Meade LB, Bearne LM, Sweeney LH, Alageel SH, Godfrey EL. Behaviour change techniques associated with adherence to prescribed exercise in patients with persistent musculoskeletal pain: Systematic review. British journal of health psychology 2019;24(1):10-30.
[60] Mogil JS. Pain genetics: past, present and future. Trends in Genetics 2012;28(6):258-266.
[61] Mogil JS. Sex differences in pain and pain inhibition: multiple explanations of a controversial phenomenon. Nat Rev Neurosci 2012;13(12):859-866.
[62] Neal BS, Barton CJ, Gallie R, O'Halloran P, Morrissey D. Runners with patellofemoral pain have altered biomechanics which targeted interventions can modify: A systematic review and meta-analysis. Gait Posture 2016;45:69-82.

[63] Neupane S, Lallukka T, Pietiläinen O, Rahkonen O, Leino-Arjas P. Trajectories of multisite musculoskeletal pain in midlife: Associations with common mental disorders. European Journal of Pain 2020;24(2):364-373.
[64] Nicholas MK, Blyth FM. Are self-management strategies effective in chronic pain treatment? Pain Management 2016;6(1):75-75–88.
[65] Øiestad BE, Juhl CB, Culvenor AG, Berg B, Thorlund JB. Knee extensor muscle weakness is a risk factor for the development of knee osteoarthritis: an updated systematic review and meta-analysis including 46 819 men and women. Br J Sports Med 2022;56(6):349-355.
[66] Oliveira CB, Maher CG, Ferreira ML, Hancock MJ, Oliveira VC, McLachlan AJ, Koes BW, Ferreira PH, Cohen SP, Pinto RZ. Epidural corticosteroid injections for lumbosacral radicular pain. Cochrane Database of Systematic Reviews 2020(4).
[67] Owen PJ, Miller CT, Mundell NL, Verswijveren SJJM, Tagliaferri SD, Brisby H, Bowe SJ, Belavy DL. Which specific modes of exercise training are most effective for treating low back pain? Network meta-analysis. British Journal of Sports Medicine 2020;54(21):1279-1287.
[68] Parreira P, Maher CG, Steffens D, Hancock MJ, Ferreira ML. Risk factors for low back pain and sciatica: an umbrella review. Spine J 2018;18(9):1715-1721.
[69] Petzke F, Klose P, Welsch P, Sommer C, Häuser W. Opioids for chronic low back pain: An updated systematic review and meta-analysis of efficacy, tolerability and safety in randomized placebo-controlled studies of at least 4 weeks of double-blind duration. European Journal of Pain 2020;24(3):497-517.
[70] Plaas H, Sudhaus S, Willburger R, Hasenbring MI. Physical activity and low back pain: the role of subgroups based on the avoidance-endurance model. Disability and Rehabilitation 2014;36(9):749-755.
[71] Rabey M, Buldo B, Duesund Helland M, Pang C, Kendell M, Beales D. Significant other interactions in people with chronic low back pain: Subgrouping and multidimensional profiles. British Journal of Pain 2021;16(3):326-340.
[72] Rabey M, Moloney N. "I Don't Know Why I've Got this Pain!" Allostasis as a Possible Explanatory Model. Phys Ther 2022;102(5).
[73] Rubinstein SM, de Zoete A, van Middelkoop M, Assendelft WJJ, de Boer MR, van Tulder MW. Benefits and harms of spinal manipulative therapy for the treatment of chronic low back pain: systematic review and meta-analysis of randomised controlled trials. BMJ 2019;364:l689.
[74] Saraceni N, Kent P, Ng L, Campbell A, Straker L, O'Sullivan P. To Flex or Not to Flex? Is There a Relationship Between Lumbar Spine Flexion During Lifting and Low Back Pain? A Systematic Review With Meta-analysis. Journal of Orthopaedic & Sports Physical Therapy 2020;50(3):121-130.
[75] Saragiotto BT, Maher CG, Yamato TP, Costa LOP, Costa LCM, Ostelo R, Macedo LG. Motor Control Exercise for Nonspecific Low Back Pain: A Cochrane Review. Spine (Phila Pa 1976) 2016;41(16):1284-1295.
[76] Schug S, Palmer G, Scott D, Alcock M, Halliwell R, Mott J, Medicine WGotAaNZCoAaFoP. Acute Pain Management: Scientific Evidence Melbourne: ANZCA & FPM, 2020.
[77] Sharma S, Abbott JH, Jensen MP. Why clinicians should consider the role of culture in chronic pain. Brazilian Journal of Physical Therapy 2018;22(5):345-346.
[78] Shiri R, Coggon D, Falah-Hassani K. Exercise for the Prevention of Low Back Pain: Systematic Review and Meta-Analysis of Controlled Trials. American Journal of Epidemiology 2017;187(5):1093-1101.
[79] Shiri R, Karppinen J, Leino-Arjas P, Solovieva S, Viikari-Juntura E. The Association Between Obesity and Low Back Pain: A Meta-Analysis. American Journal of Epidemiology 2009;171(2):135-154.
[80] Simons LE, Elman I, Borsook D. Psychological processing in chronic pain: A neural systems approach. Neuroscience & Biobehavioral Reviews 2014;39(0):61-78.

[81] Stanton TR, Moseley GL, Wong AYL, Kawchuk GN. Feeling stiffness in the back: a protective perceptual inference in chronic back pain. Scientific Reports 2017;7(1):9681.
[82] Steffens D, Maher CG, Pereira LSM, Stevens ML, Oliveira VC, Chapple M, Teixeira-Salmela LF, Hancock MJ. Prevention of Low Back Pain: A Systematic Review and Meta-analysis. JAMA Internal Medicine 2016;176(2):199-208.
[83] Struyf F, Geraets J, Noten S, Meeus M, Nijs J. A Multivariable Prediction Model for the Chronification of Non-traumatic Shoulder Pain: A Systematic Review. Pain Physician 2016;19(2):1-10.
[84] Sullivan M, Bishop S, Pivik J. The Pain Catastrophizing Scale: Development and Validation. Psychological Assessment 1995;7(4):524-532.
[85] Swain S, Coupland C, Mallen C, Kuo CF, Sarmanova A, Bierma-Zeinstra SMA, Englund M, Prieto-Alhambra D, Doherty M, Zhang W. Temporal relationship between osteoarthritis and comorbidities: a combined case control and cohort study in the UK primary care setting. Rheumatology (Oxford) 2021;60(9):4327-4339.
[86] Tegner H, Frederiksen P, Esbensen BA, Juhl C. Neurophysiological Pain Education for Patients With Chronic Low Back Pain: A Systematic Review and Meta-Analysis. The Clinical Journal of Pain 2018;34(8).
[87] Vambheim SM, Kyllo TM, Hegland S, Bystad M. Relaxation techniques as an intervention for chronic pain: A systematic review of randomized controlled trials. Heliyon 2021;7(8):e07837-e07837.
[88] Van Looveren E, Bilterys T, Munneke W, Cagnie B, Ickmans K, Mairesse O, Malfliet A, De Baets L, Nijs J, Goubert D, Danneels L, Moens M, Meeus M. The Association between Sleep and Chronic Spinal Pain: A Systematic Review from the Last Decade. J Clin Med 2021;10(17).
[89] Vibe Fersum K, O'Sullivan P, Skouen JS, Smith A, Kvåle A. Efficacy of classification-based cognitive functional therapy in patients with non-specific chronic low back pain: A randomized controlled trial. European Journal of Pain 2013;17(6):916-928.
[90] Vicenzino B, Paungmali A, Teys P. Mulligan's mobilization-with-movement, positional faults and pain relief: current concepts from a critical review of literature. Man Ther 2007;12(2):98-108.
[91] Waddell G, Newton M, Henderson I, Somerville D, Main CJ. A Fear-Avoidance Beliefs Questionnaire (FABQ) and the role of fear-avoidance beliefs in chronic low back pain and disability. PAIN 1993;52(2):157-168.
[92] Watson JA, Ryan CG, Cooper L, Ellington D, Whittle R, Lavender M, Dixon J, Atkinson G, Cooper K, Martin DJ. Pain Neuroscience Education for Adults With Chronic Musculoskeletal Pain: A Mixed-Methods Systematic Review and Meta-Analysis. The Journal of Pain 2019;20(10):1140.e1141-1140.e1122.
[93] Wertli MM, Rasmussen-Barr E, Held U, Weiser S, Bachmann LM, Brunner F. Fear-avoidance beliefs-a moderator of treatment efficacy in patients with low back pain: a systematic review. Spine J 2014;14(11):2658-2678.
[94] Williams ACC, Fisher E, Hearn L, Eccleston C. Psychological therapies for the management of chronic pain (excluding headache) in adults. Cochrane Database Syst Rev 2020;8(8):Cd007407.
[95] Zale EL, Maisto SA, Ditre JW. Interrelations between pain and alcohol: An integrative review. Clin Psychol Rev 2015;37:57-71.
[96] Zheng H, Chen C. Body mass index and risk of knee osteoarthritis: systematic review and meta-analysis of prospective studies. BMJ Open 2015;5(12):e007568.

Full results tables

Respondents' understanding of pain (n=1403). Data are presented as n (%).

Belief Statement	Full cohort (n=1403)			Pain subgroup (n=1185)			No pain subgroup (n=218)			*p*-value*
	True	False	Unsure	True	False	Unsure	True	False	Unsure	
Pain only occurs when you are injured or at risk of being injured	120 (8.6)	1194 (85.1)	89 (6.3)	88 (7.4)	1019 (86.0)	78 (6.6)	32 (14.7)	175 (80.3)	11 (5.0)	.002
Persistent pain means that an injury hasn't healed properly	354 (25.2)	819 (58.4)	230 (16.4)	293 (24.7)	700 (59.1)	192 (16.2)	61 (28.0)	119 (54.6)	38 (17.4)	.45
Pain always occurs when you are injured	214 (15.2)	1050 (74.8)	139 (9.9)	184 (15.5)	884 (74.6)	117 (9.9)	30 (13.8)	166 (76.2)	22 (10.1)	.80
When you injure yourself, the environment that you are in will not affect the amount of pain you experience, as long as the injury is exactly the same	197 (14.0)	911 (64.9)	295 (21.0)	168 (14.2)	759 (64.0)	258 (21.8)	29 (13.3)	152 (69.7)	37 (17.0)	.22
The brain decides when you will experience pain	852 (60.7)	280 (20.0)	271 (19.3)	713 (60.2)	242 (20.4)	230 (19.4)	139 (63.8)	38 (17.4)	41 (18.8)	.54
There is always a simple explanation for why someone has pain	119 (8.5)	1165 (83.0)	119 (8.5)	104 (8.8)	977 (82.4)	104 (8.8)	15 (6.9)	188 (86.2)	15 (6.9)	.39
Most pain gets better	514 (36.6)	629 (44.8)	260 (18.5)	402 (33.9)	563 (47.5)	220 (18.6)	112 (51.4)	66 (30.3)	40 (18.4)	**<.0001**

Once you have pain, you're always likely to have pain	144 (10.3)	1006 (71.7)	253 (18.0)	132 (11.1)	828 (69.9)	225 (19.0)	12 (5.5)	178 (81.6)	28 (12.8)	.001
More pain means more tissue damage (i.e., damage to joints, nerves, tendons or muscles)	368 (26.2)	644 (45.9)	391 (27.9)	316 (26.7)	531 (44.8)	338 (28.5)	52 (23.8)	113 (51.8)	53 (24.3)	.16
There is always tissue damage to explain pain	85 (6.1)	996 (71.0)	322 (23.0)	72 (6.1)	837 (70.6)	276 (23.3)	13 (6.0)	159 (72.9)	46 (21.1)	.77
It is possible to have tissue damage but no pain	830 (59.2)	113 (8.0)	460 (32.8)	685 (57.8)	95 (8.0)	405 (34.2)	145 (66.5)	18 (8.3)	55 (25.2)	.03
Findings on scans like arthritis and disc bulges are always associated with pain	436 (31.1)	597 (42.6)	370 (26.4)	380 (32.1)	498 (42.0)	307 (25.9)	57 (25.7)	99 (45.4)	63 (28.9)	.17
An increase in pain is an indication that you should stop doing what you're doing until the pain decreases	748 (53.3)	448 (31.9)	207 (14.8)	627 (52.9)	383 (32.3)	175 (14.8)	121 (55.5)	65 (29.8)	32 (14.7)	.74
Pain may mean something is out of place	1,067 (76.0)	222 (15.8)	114 (8.1)	884 (74.6)	200 (16.9)	101 (8.5)	183 (83.9)	22 (10.1)	13 (6.0)	.01
If pain is not associated with injury or tissue damage it must be psychological	167 (11.9)	933 (66.5)	303 (21.6)	134 (11.3)	801 (67.6)	250 (21.1)	33 (15.1)	132 (60.6)	53 (24.3)	.10
Pain is abnormal	333 (23.7)	909 (64.8)	161 (11.5)	280 (23.6)	766 (64.6)	139 (11.7)	53 (24.3)	143 (65.6)	22 (10.1)	.78
Pain means you aren't healthy	75 (5.4)	1229 (87.6)	99 (7.1)	60 (5.1)	1043 (88.0)	82 (6.9)	15 (6.9)	186 (85.3)	17 (7.8)	.47
Pain is usually caused by physical overuse or excessive strain	367 (26.2)	849 (60.5)	187 (13.3)	304 (25.6)	729 (61.5)	152 (12.8)	63 (28.9)	120 (55.0)	35 (16.1)	.18
Pain is usually caused by work or by an accident at work	92 (6.6)	1201 (85.6)	110 (7.8)	78 (6.6)	1021 (86.2)	86 (7.3)	14 (6.4)	180 (82.6)	24 (11.0)	.17

Full pain relief is necessary before returning to work, sport or other daily activities	171 (12.2)	1078 (76.8)	154 (11.0)	149 (12.6)	903 (76.2)	133 (11.2)	22 (10.1)	175 (80.3)	21 (9.6)	.42
Tests like MRI scans, x-rays and ultrasound imaging are critical to identify the source of pain	650 (46.3)	480 (34.2)	273 (19.5)	565 (47.7)	401 (33.8)	219 (18.5)	85 (39.0)	79 (36.2)	54 (24.8)	.03
The source of pain must always be identified for adequate pain treatment to occur	838 (59.7)	401 (28.6)	164 (11.7)	712 (60.1)	334 (28.2)	139 (11.7)	126 (57.8)	67 (30.7)	25 (11.5)	.74
It is possible to have the right treatment for pain without having tests like MRI scans, x-rays or ultrasound imaging	921 (65.7)	194 (13.8)	288 (20.5)	764 (64.5)	176 (14.8)	245 (20.7)	157 (72.0)	18 (8.3)	43 (19.7)	.02
When I have pain I think to myself "don't make such a fuss"	1,081 (77.0)	253 (18.0)	69 (4.9)	928 (78.3)	202 (17.0)	55 (4.6)	153 (70.2)	51 (23.4)	14 (6.4)	.03
When I have pain I carry on doing what I'm doing no matter what	837 (59.7)	436 (31.1)	130 (9.3)	742 (62.6)	346 (29.2)	97 (8.2)	95 (43.6)	90 (41.3)	33 (15.1)	**<.0001**
When I am in pain my family/partner should look after me	164 (11.7)	1075 (76.6)	164 (11.7)	123 (10.4)	925 (78.1)	137 (11.6)	41 (18.8)	150 (68.8)	27 (12.4)	.001

*p-value reflects difference between pain and no pain subgroups.

Respondents' understanding of pain treatments. Data are presented as n (%).

Statement	Full cohort (n=1209)			Pain subgroup (n=1022)			No pain subgroup (n=187)			*p*-value*
	True	False	Unsure	True	False	Unsure	True	False	Unsure	
It is important to rest when you have pain	492 (40.7)	471 (39.0)	246 (20.4)	415 (40.6)	413 (40.4)	194 (19.0)	77 (41.2)	58 (31.0)	52 (27.8)	.01
It is important to stay active when you have pain	771 (63.8)	178 (14.7)	260 (21.5)	665 (65.1)	147 (14.4)	210 (20.6)	106 (56.7)	31 (16.6)	50 (26.7)	.08
It is important to gradually increase your activity when you have pain	819 (67.7)	163 (13.5)	227 (18.8)	702 (68.7)	132 (12.9)	188 (18.4)	117 (62.6)	31 (16.6)	39 (20.9)	.23
If you experience pain, you should just keep pushing through	189 (15.6)	777 (64.3)	243 (20.1)	170 (16.6)	642 (62.8)	210 (20.6)	19 (10.2)	135 (72.2)	33 (17.6)	.03
It is possible to manage pain well yourself	824 (68.2)	192 (15.9)	193 (16.0)	683 (66.8)	178 (17.4)	161 (15.8)	141 (75.4)	14 (7.5)	32 (17.1)	.003
It is important to seek professional advice for pain care	867 (71.7)	158 (13.1)	184 (15.2)	739 (72.3)	130 (12.7)	153 (15.0)	128 (68.4)	28 (15.0)	31 (16.6)	.55
It is important to seek treatments (medications, injections, surgery, hands-on treatments) from professionals to get pain relief	737 (61.0)	280 (23.2)	192 (15.9)	624 (61.1)	237 (23.2)	161 (15.8)	113 (60.4)	43 (23.0)	31 (16.6)	.96
Medications can be helpful for treating pain	1136 (94.0)	22 (1.8)	51 (4.2)	958 (93.7)	19 (1.9)	45 (4.4)	178 (95.2)	3 (1.6)	6 (3.2)	.73
Medications are always helpful for treating pain	144 (11.9)	939 (77.7)	126 (10.4)	125 (12.2)	784 (76.7)	113 (11.1)	19 (10.2)	155 (82.9)	13 7.0)	.14
Medications are never necessary for treating pain	64 (5.3)	1062 (87.8)	83 (6.9)	54 (5.3)	897 (87.8)	71 (7.0)	10 (5.4)	165 (88.2)	12 (6.2)	.97
Stronger medications are always better for pain relief	141 (11.7)	858 (71.0)	210 (17.4)	121 (11.8)	720 (70.4)	181 (17.7)	20 (10.7)	138 (73.8)	29 (15.5)	.65
Injections can be helpful for treating pain	983 (81.3)	53 (4.4)	173 (14.3)	828 (81.0)	44 (4.3)	150 (14.7)	155 (82.9)	9 (4.8)	23 (12.3)	.68
Injections are always helpful for treating pain	156 (12.9)	762 (63.0)	291 (24.1)	137 (13.4)	636 (62.2)	249 (24.4)	19 (10.2)	126 (67.4)	42 (22.5)	.33

Injections are never necessary for treating pain	70 (5.8)	963 (79.6)	176 (14.6)	58 (5.7)	812 (79.4)	152 (14.9)	12 (6.4)	151 (80.8)	24 (12.8)	.73
Surgery can be helpful for treating pain	998 (82.6)	39 (3.2)	172 (14.2)	834 (81.6)	36 (3.5)	152 (14.9)	164 (87.7)	3 (1.6)	20 (10.7)	.11
Surgery is always helpful for treating pain	37 (3.1)	971 (80.3)	201 (16.6)	34 (3.3)	810 (79.3)	178 (17.4)	3 (1.6)	161 (86.1)	23 (12.3)	.08
Surgery is never necessary for treating pain	36 (3.0)	989 (81.8)	184 (15.2)	32 (13.1)	833 (81.5)	157 (15.4)	4 (2.1)	156 (83.4)	27 (14.4)	.71
Surgery should only be considered as a final option when other treatments have not worked	927 (76.7)	140 (11.6)	142 (11.8)	794 (77.7)	118 (11.6)	110 (10.8)	133 (71.1)	22 (11.8)	32 (17.1)	.04
There is always some surgical procedure or medication that will get rid of pain	81 (6.7)	980 (81.1)	148 (12.2)	72 (7.0)	822 (80.4)	128 (12.5)	9 (4.8)	158 (84.5)	20 (10.7)	.38
Exercise can be helpful for treating pain	1103 (91.2)	29 (2.4)	77 (6.4)	931 (91.1)	28 (2.7)	63 (6.2)	172 (92.0)	1 (0.5)	14 (7.5)	.16
Exercise is always helpful for treating pain	145 (12.0)	836 (69.1)	228 (18.9)	127 (12.4)	696 (68.1)	199 (19.5)	18 (9.6)	140 (74.9)	29 (15.5)	.18
Exercise is never necessary for treating pain	35 (2.9)	1049 (86.8)	125 (10.3)	31 (3.0)	886 (86.7)	105 (10.3)	4 (2.1)	163 (87.2)	20 (10.7)	.79
You should be very careful exercising when you have pain	1090 (90.2)	50 (4.1)	69 (5.7)	923 (90.3)	42 (4.1)	57 (5.6)	167 (89.3)	8 (4.3)	12 (6.4)	.89
Stretching is always an effective exercise for pain	458 (37.9)	479 (39.6)	272 (22.5)	402 (39.3)	397 (38.8)	223 (21.8)	56 (30.0)	82 (43.8)	49 (26.2)	.05
Good core stability is key to managing pain	620 (51.3)	234 (19.3)	355 (29.4)	547 (53.5)	118 (18.4)	287 (28.1)	73 (39.0)	46 (24.6)	68 (36.4)	.001
It is always important to maintain good alignment when exercising, especially if you have pain	1037 (85.8)	39 (3.2)	133 (11.0)	871 (85.2)	35 (3.4)	116 (11.4)	166 (88.8)	4 (2.1)	17 (9.1)	.41
Good advice can be sufficient pain care	627 (51.9)	359 (29.7)	223 (18.4)	516 (50.5)	322 (31.5)	184 (18.0)	111 (59.4)	37 (19.8)	39 (20.9)	.005
Understanding how pain works is an effective pain treatment	773 (63.9)	215 (17.8)	221 (18.3)	650 (63.6)	188 (18.4)	184 (18.0)	123 (65.8)	27 (14.4)	37 (19.8)	.41
Relaxation and mental distraction are good ways of treating pain	922 (76.3)	114 (9.4)	173 (14.3)	771 (75.4)	108 (10.6)	143 (14.0)	151 (80.8)	6 (3.2)	30 (16.0)	.01

Psychological treatments (talk therapies, stress management, mindfulness) can be helpful for treating pain	835 (69.1)	136 (11.2)	238 (19.7)	684 (66.9)	130 (12.7)	208 (20.4)	151 (80.8)	6 (3.2)	30 (16.0)	**.0001**
Psychological treatments are always helpful for treating pain	121 (10.0)	778 (64.3)	310 (25.6)	102 (10.0)	653 (63.9)	267 (26.1)	19 (10.2)	125 (66.8)	43 (23.0)	.66
Psychological treatments are never necessary for treating pain	71 (5.9)	851 (70.4)	287 (23.7)	63 (6.2)	709 (69.4)	250 (24.5)	8 (4.3)	142 (75.9)	37 (19.8)	.18
Psychological treatments should only be used for pain relief when nothing else has worked	108 (8.9)	818 (67.7)	283 (23.4)	90 (8.8)	684 (66.9)	248 (24.3)	18 (9.6)	134 (71.7)	35 (18.7)	.26
Addressing mood and stress/anxiety is important for good pain care	983 (81.3)	77 (6.4)	149 (12.3)	823 (80.5)	68 (6.6)	131 (12.8)	160 (85.6)	9 (4.8)	18 (9.6)	.27
'Hands-on' therapies (massage, manipulation) can be helpful for treating pain	1126 (93.1)	24 (2.0)	59 (4.9)	945 (92.5)	24 (2.4)	53 (5.2)	181 (96.8)	0 (0.0)	6 (3.2)	.05
'Hands-on' therapies are always helpful for treating pain	165 (13.7)	855 (70.7)	189 (15.6)	145 (14.2)	712 (69.7)	165 (16.1)	20 (10.7)	143 (76.5)	24 (12.8)	.17
'Hands-on' therapies are never necessary for treating pain	40 (3.3)	1028 (85.0)	141 (11.7)	33 (3.2)	861 (84.2)	128 (12.5)	7 (3.7)	167 (89.3)	13 (7.0)	.09
Physical therapies (physiotherapy, osteopathy, chiropractic) should always include 'hands-on' treatments for pain relief	408 (33.8)	470 (38.9)	331 (27.4)	361 (35.2)	395 (38.6)	266 (26.0)	47 (25.1)	75 (40.1)	65 (34.8)	.01
It is important to treat underlying lifestyle factors for pain relief (e.g. sleep, stress, work habits, exercise, diet)	1085 (89.7)	26 (2.1)	98 (8.1)	913 (89.3)	22 (2.2)	87 (8.5)	172 (92.0)	4 (2.1)	11 (5.9)	.48
I am usually willing to change my habits and behaviours to improve my health and pain care	1064 (88.0)	66 (5.5)	79 (6.5)	899 (88.0)	56 (5.5)	67 (6.6)	165 (88.2)	10 (5.4)	12 (6.4)	.99
Future episodes of pain can be reduced or avoided by avoiding aggravating activities	934 (77.2)	138 (11.4)	137 (11.3)	728 (76.5)	125 (12.2)	115 (11.2)	152 (81.3)	13 (7.0)	22 (11.8)	.11
Future episodes of pain can be reduced or avoided through exercise	697 (57.7)	248 (20.5)	264 (21.8)	568 (55.6)	222 (21.7)	232 (22.7)	129 (69.0)	26 (13.9)	32 (17.1)	.003
Future episodes of pain can be reduced or avoided by getting regular 'hands-on' treatments like massage or manipulation	645 (53.3)	254 (21.0)	310 (25.6)	534 (52.2)	228 (22.3)	260 (25.4)	111 (59.4)	26 (13.9)	50 (26.7)	.03

Future episodes of pain can be reduced or avoided by addressing lifestyle factors like sleep, weight and stress	969 (80.1)	101 (8.3)	139 (11.5)	808 (79.1)	95 (9.3)	119 (11.6)	161 (86.1)	6 (3.2)	20 (10.7)	.02
Future episodes of pain cannot be avoided	496 (41.0)	459 (38.0)	254 (21.0)	448 (43.8)	369 (36.1)	205 (20.1)	48 (25.7)	90 (48.1)	49 (26.2)	**<.0001**

**p*-value reflects difference between pain and no pain subgroups.

Respondents' beliefs about factors influencing pain (n=1145). Data are presented as n (%).

Influencing factor	Full cohort (n=1145)	Pain subgroup (n=970)	No pain subgroup (n=175)	*p*-value*
		Yes, n(%)		
Mood	705 (61.6)	588 (60.6)	117 (66.9)	.12
Beliefs about injury and tissue damage	478 (41.8)	381 (39.3)	97 (55.4)	**.0001**
Posture and alignment (e.g., spinal posture, leg alignment, foot posture)	1048 (91.5)	880 (90.7)	168 (96.0)	.02
Sleep	821 (71.7)	688 (70.9)	133 (76.0)	.17
Diet	715 (62.4)	596 (61.4)	119 (68.0)	.10
Weak muscles	915 (79.9)	767 (79.1)	148 (84.6)	.10
Muscle tightness	925 (80.8)	776 (80.0)	149 (85.1)	.11
Age	864 (75.5)	731 (75.4)	133 (76.0)	.86
Social support (support / lack of support from family, friends, colleagues, others)	328 (28.7)	266 (27.4)	62 (35.4)	.03
How you think about pain	606 (52.9)	499 (51.4)	107 (61.1)	.02
Weight	984 (85.9)	822 (84.7)	162 (92.6)	.01
Stress (at home, at work etc)	820 (71.6)	684 (70.5)	136 (77.7)	.05
Access to appropriate healthcare	603 (52.7)	496 (51.1)	107 (61.1)	.02

Ergonomics (e.g., work set up and practices)	764 (66.7)	628 (64.7)	136 (77.7)	.001
Culture (society's beliefs and practices)	268 (23.4)	209 (21.6)	59 (33.7)	.0005
Amount of tissue damage or injury (e.g., disc bulges, arthritis, tendon, muscle, ligament strains)	1047 (91.4)	886 (91.3)	161 (92.0)	.77
Whether you are male or female	209 (18.2)	164 (16.9)	45 (25.7)	.01
Education level	119 (10.4)	89 (9.2)	30 (17.1)	.001
Other health problems (e.g. heart disease, diabetes, lung conditions etc)	731 (63.8)	609 (62.8)	122 (69.7)	.08
Alcohol or drug use	639 (55.8)	520 (53.6)	119 (68.0)	.0004
Genetics	656 (57.3)	537 (55.4)	119 (68)	.002
None of the above	11 (1.0)	4 (0.4)	2 (1.1)	.22

*p-value reflects difference between pain and no pain subgroups.

Respondents' beliefs about physical activity and pain (n=1122). Data are presented as n (%).

	Full cohort (n=1122)							Pain cohort (n=951)							No pain cohort (n=171)							**p-value***
	STD	D	SMD	N	SMA	A	SA	STD	D	SMD	N	SMA	A	SA	STD	D	SMD	N	SMA	A	STA	
Physical activity makes pain worse	97 (8.7)	202 (18.0)	196 (17.5)	318 (28.3)	201 (17.9)	66 (5.9)	42 (3.7)	80 (8.4)	163 (17.1)	163 (17.1)	266 (28.0)	177 (18.6)	61 (6.4)	41 (4.3)	17 (9.9)	39 (22.8)	33 (19.3)	52 (30.4)	24 (14.0)	5 (2.9)	1 (0.6)	.03
Physical activity might harm my body if I am in pain	43 (3.8)	160 (14.3)	143 (12.8)	190 (16.9)	361 (32.2)	182 (16.2)	43 (3.8)	35 (3.7)	138 (14.5)	122 (12.8)	159 (16.7)	310 (32.6)	151 (15.9)	36 (3.8)	8 (4.7)	22 (12.9)	21 (12.3)	31 (18.1)	51 (29.8)	31 (18.1)	7 (4.1)	.95
I should not do physical activities which (might) make pain worse	39 (3.5)	137 (12.2)	170 (15.1)	157 (14.0)	268 (23.9)	261 (23.3)	90 (8.0)	36 (3.8)	123 (12.9)	138 (14.5)	133 (14.0)	225 (23.7)	223 (23.4)	73 (7.7)	3 (1.8)	14 (8.2)	32 (18.7)	24 (14.0)	43 (25.2)	38 (22.2)	17 (9.9)	.30

STD: Strongly disagree; D: Disagree: SMD: Somewhat disagree; N: neither agree nor disagree; SMA: somewhat agree; A: Agree; STA: Strongly agree
Questions taken from the Fear-Avoidance Beliefs Questionnaire [92]. **p*-value reflects difference between pain and no pain subgroups.

Results from the Pain Catastrophising Scale including comparisons between the pain subgroup and the no pain subgroup **(n=1110). Data are presented as n (%) unless otherwise specified.**

Individual Statements When I'm in pain….	Full cohort (n= 1110)					Pain subgroup (n=941)					No pain subgroup (n=169)					*p*-value*
	Not at all	A little	Moderate amount	A lot	A great deal	Not at all	A little	Moderate amount	A lot	A great deal	Not at all	A little	Moderate amount	A lot	A great deal	
I worry all the time about whether the pain will end	131 (11.8)	384 (34.6)	300 (27.0)	180 (16.2)	115 (10.4)	103 (11.0)	309 (32.8)	263 (28.0)	162 (17.2)	104 (11.0)	28 (16.6)	75 (44.4)	37 (21.9)	18 (10.6)	11 (6.5)	.001
I feel I can't go on	448 (40.4)	325 (29.3)	187 (16.9)	98 (8.8)	52 (4.7)	356 (37.8)	279 (29.6)	170 (18.1)	87 (9.2)	49 (5.2)	92 (54.4)	46 (27.2)	17 (10.1)	11 (6.5)	3 (1.2)	.0004
It's terrible and I think it's never going to get any better	330 (29.7)	370 (33.3)	185 (16.7)	142 (12.8)	83 (7.5)	241 (25.6)	322 (34.2)	167 (17.8)	132 (14.0)	79 (8.4)	89 (52.7)	48 (28.4)	18 (10.6)	10 (5.9)	4 (2.4)	**<.0001**
It's awful and I feel that it overwhelms me	324 (29.2)	375 (33.8)	199 (17.9)	139 (12.5)	73 (6.6)	252 (26.8)	311 (33.0)	180 (19.1)	128 (13.6)	70 (7.4)	71 (42.6)	64 (37.9)	19 (11.2)	11 (6.5)	3 (1.8)	**<.0001**
I feel I can't stand it anymore	352 (31.7)	379 (34.1)	180 (16.2)	120 (10.8)	79 (7.1)	272 (28.9)	314 (33.4)	168 (17.8)	112 (11.9)	75 (8.0)	80 (47.3)	65 (38.5)	12 (7.1)	8 (4.7)	4 (2.4)	**<.0001**
I become afraid that the pain will get worse	186 (16.8)	439 (39.6)	228 (20.5)	165 (14.9)	92 (8.3)	147 (15.6)	355 (37.7)	198 (21.0)	152 (16.2)	89 (9.5)	39 (23.1)	84 (49.7)	30 (17.8)	13 (7.7)	3 (1.8)	**<.0001**

I keep thinking of other painful events	668 (60.2)	266 (24.0)	100 (9.0)	50 (4.5)	26 (2.3)	551 (58.6)	230 (24.4)	87 (9.2)	48 (5.1)	25 (2.7)	117 (69.2)	36 (21.3)	13 (7.7)	2 (1.2)	1 (0.6)	.02
I anxiously want the pain to go away	201 (18.1)	380 (34.2)	194 (17.5)	201 (18.1)	134 (12.1)	163 (17.3)	308 (32.7)	163 (17.3)	182 (19.3)	125 (13.3)	38 (22.5)	72 (42.6)	31 (18.3)	19 (11.2)	9 (5.3)	.001
I can't seem to keep it out of my mind	208 (18.7)	404 (36.4)	224 (20.2)	188 (16.9)	86 (7.8)	173 (18.4)	331 (35.2)	192 (20.4)	165 (17.5)	80 (8.5)	35 (20.7)	73 (43.2)	32 (18.9)	23 (13.6)	6 (3.6)	.06
I keep thinking about how much it hurts	138 (12.4)	463 (41.7)	250 (22.5)	177 (16.0)	82 (7.4)	118 (12.5)	378 (40.2)	211 (22.4)	157 (16.7)	77 (8.2)	20 (11.8)	85 (50.3)	39 (23.1)	20 (11.8)	5 (3.0)	.03
I keep thinking about how badly I want the pain to stop	136 (12.2)	410 (36.9)	220 (19.8)	203 (18.3)	141 (12.7)	112 (11.9)	336 (35.7)	183 (19.4)	178 (18.9)	132 (14.0)	24 (14.2)	74 (43.8)	37 (21.9)	25 (14.8)	9 (5.3)	.01
There's nothing I can do to reduce the intensity of the pain	304 (27.4)	395 (35.6)	241 (21.7)	113 (10.2)	57 (5.1)	231 (24.6)	335 (35.6)	215 (22.8)	107 (11.4)	53 (5.6)	73 (43.2)	60 (35.5)	26 (15.4)	6 (3.6)	4 (2.4)	<.001
I wonder whether something serious may happen	345 (31.1)	427 (38.5)	170 (15.3)	101 (9.1)	67 (6.0)	293 (31.1)	354 (37.6)	144 (15.3)	88 (9.4)	62 (6.6)	52 (30.8)	73 (43.2)	26 (15.4)	13 (7.7)	5 (3.0)	.32
Magnification subscale score, range 0-12, median (IQR) (min,max)			3 (1, 5) (0, 12)					3 (1, 5) (0, 12)					2 (1, 4) (0, 9)			.0004
Rumination subscale score, range 0-16, median (IQR) (min,max)			6 (4, 10) (0, 16)					6 (4, 10) (0, 16)					5 (3,8) (0,16)			.001

Helplessness subscale score, range 0-24, median (IQR) (min,max)	7 (3, 12) (0, 24)	7 (4, 12) (0, 24)	4 (2, 7) (0, 24)	**<.0001**
Total Pain Catastrophising Scale score, range 0-52, median (IQR) (min,max)	16 (9, 26) (0, 52)	17 (9, 27) (0, 52)	12 (7, 19) (0, 48)	**<.0001**
Total score ≥30	214 (19.3)	200 (21.2)	14 (8.3)	**<.0001**

**p*-value reflects difference between pain and no pain subgroups.

Factors influencing healthcare decision making (n=988).

Decision making influenced by:	Full cohort (n=988) Ranking, n (%)										
	1	2	3	4	5	6	7	8	9	10	n/a
Recommendations from friends / family	105 (10.6)	73 (7.4)	123 (12.4)	91 (9.2)	69 (7.0)	69 (7.0)	57 (5.8)	49 (5.0)	48 (4.9)	35 (3.5)	269 (27.2)
Recommendations from my doctor	442 (44.7)	164 (16.6)	73 (7.4)	49 (5.0)	42 (4.2)	32 (3.2)	19 (1.9)	17 (1.7)	25 (2.5)	20 (2.0)	105 (10.6)
Recommendations from my other registered health professional	155 (15.7)	377 (38.2)	124 (12.6)	43 (4.3)	44 (4.4)	26 (2.6)	32 (3.2)	17 (1.7)	23 (2.3)	10 (1.0)	137 (13.9)
Recommendations from my alternative/complementary therapist	22 (2.2)	61 (6.2)	139 (14.1)	127 (12.9)	56 (5.7)	40 (4.0)	42 (4.2)	42 (4.2)	39 (4.0)	46 (4.7)	374 (37.9)
General internet search	37 (3.7)	47 (4.8)	92 (9.3)	130 (13.2)	133 (13.5)	70 (7.1)	64 (6.5)	71 (7.2)	47 (4.8)	46 (4.7)	251 (25.4)
I look for treatments that have a lot of scientific evidence	33 (3.3)	55 (5.6)	94 (9.5)	124 (12.6)	144 (14.6)	146 (14.8)	61 (6.2)	44 (4.4)	26 (2.6)	16 (1.6)	245 (24.8)
I research treatments on websites like Cochrane, Pedro, NHS Choices, NICE guidelines	23 (2.3)	30 (3.0)	74 (7.5)	89 (9.0)	91 (9.2)	122 (12.3)	119 (12.0)	61 (6.2)	40 (4.0)	36 (3.6)	303 (30.7)
I look for treatments that seem easy and are likely to have fast effects	23 (2.3)	29 (2.9)	69 (7.0)	88 (8.9)	87 (8.8)	113 (11.4)	133 (13.5)	139 (14.1)	53 (5.4)	27 (2.7)	227 (23.0)
I look for treatments that are least invasive	34 (3.4)	52 (5.3)	45 (4.6)	69 (7.0)	102 (10.3)	88 (8.9)	113 (11.4)	126 (12.8)	137 (13.9)	43 (4.3)	179 (18.1)
I look for treatments where I can take control and learn how to self-manage	91 (9.2)	60 (6.1)	93 (9.4)	90 (9.1)	91 (9.2)	94 (9.5)	79 (8.0)	67 (6.8)	96 (9.7)	96 (9.7)	131 (13.3)

Decision making influenced by:	Pain subgroup (n=835) Ranking, n(%)										
	1	2	3	4	5	6	7	8	9	10	n/a
Recommendations from friends / family	87 (10.4)	67 (8.0)	98 (11.7)	79 (9.5)	59 (7.1)	56 (6.7)	45 (5.4)	40 (4.8)	38 (4.6)	29 (3.5)	237 (28.4)
Recommendations from my doctor	380 (45.5)	140 (16.8)	62 (7.4)	41 (4.9)	34 (4.1)	26 (3.1)	15 (1.8)	13 (1.6)	23 (2.8)	15 (1.8)	86 (10.3)

Recommendations from my other registered health professional	130 (15.6)	316 (37.8)	116 (13.9)	37 (4.4)	33 (4.0)	20 (2.4)	28 (3.4)	14 (1.7)	18 (2.2)	9 (1.1)	114 (13.6)
Recommendations from my alternative/complementary therapist	21 (2.5)	54 (6.5)	118 (14.1)	107 (12.8)	48 (5.8)	33 (4.0)	32 (3.8)	32 (3.8)	31 (3.7)	37 (4.4)	322 (38.6)
General internet search	29 (3.5)	40 (4.8)	79 (9.5)	108 (12.9)	110 (13.2)	60 (7.2)	52 (6.2)	60 (7.2)	43 (5.2)	33 (4.0)	221 (26.5)
I look for treatments that have a lot of scientific evidence	27 (3.2)	46 (5.5)	81 (9.7)	105 (12.6)	123 (14.7)	116 (13.9)	52 (6.2)	39 (4.7)	26 (3.1)	14 (1.7)	206 (24.7)
I research treatments on websites like Cochrane, Pedro, NHS Choices, NICE guidelines	19 (2.3)	24 (2.9)	61 (7.3)	71 (8.5)	79 (9.5)	107 (12.8)	99 (11.9)	51 (6.1)	34 (4.1)	31 (3.7)	259 (31.0)
I look for treatments that seem easy and are likely to have fast effects	21 (2.5)	21 (2.5)	58 (7.0)	73 (8.7)	72 (8.6)	101 (12.1)	118 (14.1)	115 (13.8)	40 (4.8)	21 (2.5)	195 (23.4)
I look for treatments that are least invasive	29 (3.5)	40 (4.8)	34 (4.1)	63 (7.5)	89 (10.7)	75 (9.0)	98 (11.7)	108 (12.9)	110 (13.2)	37 (4.4)	152 (18.2)
I look for treatments where I can take control and learn how to self-manage	73 (8.7)	55 (6.6)	79 (9.5)	78 (9.3)	79 (9.5)	80 (9.6)	63 (7.5)	56 (6.7)	81 (9.7)	83 (9.9)	108 (12.9)

Decision making influenced by:	No pain subgroup (n=153) Ranking, n(%)											*p*-value*
	1	2	3	4	5	6	7	8	9	10	n/a	
Recommendations from friends / family	18 (11.8)	6 (3.9)	25 (16.3)	12 (7.8)	10 (6.5)	13 (8.5)	12 (7.8)	9 (5.9)	10 (6.5)	6 (3.9)	32 (20.9)	.30
Recommendations from my doctor	62 (40.5)	24 (15.7)	11 (7.2)	8 (5.2)	8 (5.2)	6 (3.9)	4 (2.6)	4 (2.6)	2 (1.3)	5 (3.3)	19 (12.4)	.83
Recommendations from my other registered health professional	25 (16.3)	61 (39.9)	8 (5.2)	6 (3.9)	11 (7.2)	6 (3.9)	4 (2.6)	3 (2.0)	5 (3.3)	1 (0.6)	23 (15.0)	.19
Recommendations from my alternative/complementary therapist	1 (0.6)	7 (4.6)	21 (13.7)	20 (13.1)	8 (5.2)	7 (4.6)	10 (6.5)	10 (6.5)	8 (5.2)	9 (5.9)	52 (34.0)	.49
General internet search	8 (5.2)	7 (4.6)	13 (8.5)	22 (14.4)	23 (15.0)	10 (6.5)	12 (7.8)	11 (7.2)	4 (2.6)	13 (8.5)	30 (19.6)	.27
I look for treatments that have a lot of scientific evidence	6 (3.9)	9 (5.9)	13 (8.5)	19 (12.4)	21 (13.7)	30 (19.6)	9 (5.9)	5 (3.3)	0 (0.0)	2 (1.3)	39 (25.5)	.54
I research treatments on websites like Cochrane, PEDro, NHS Choices, NICE guidelines	4 (2.6)	6 (3.9)	13 (8.5)	18 (11.8)	12 (7.8)	15 (9.8)	20 (13.1)	10 (6.5)	6 (3.9)	5 (3.3)	44 (28.8)	.94
I look for treatments that seem easy and are likely to have fast effects	2 (1.3)	8 (5.2)	11 (7.2)	15 (9.8)	15 (9.8)	12 (7.8)	15 (9.8)	24 (15.7)	13 (8.5)	6 (3.9)	32 (20.9)	.21

I look for treatments that are least invasive	5 (3.3)	12 (7.8)	11 (7.2)	6 (3.9)	13 (8.5)	13 (8.5)	15 (9.8)	18 (11.8)	27 (17.6)	6 (3.9)	27 (17.6)	.39
I look for treatments where I can take control and learn how to self-manage	18 (11.8)	5 (3.3)	14 (9.2)	12 (7.8)	12 (7.8)	14 (9.2)	16 (10.5)	11 (7.2)	15 (9.8)	13 (8.5)	23 (15.0)	.77

PEDro: Physiotherapy Evidence Database; NHS: National Health Service (UK): NICE: National Institute of Clinical Excellence
**p*-value reflects difference between pain and no pain subgroups

Guernsey sociodemographic data (2019-2020) to facilitate comparison with other jurisdictions.

Item		Metric	Date
Population		62,706	March 2019[1]
Expenditure on health		£177m (29%)	2018[1]
Life expectancy at birth		82.7 years	2017-2019[1]
Gross domestic product per capita		£51,868	2019[2]
Median earnings		£33,530	4th quarter, 2019[1]
Mean property price		£441,295	4th quarter, 2019[1]
Percentage of population with private health insurance		50%	2018[3]
Country of birth (Percentage of total population)	Guernsey	52.9	March 2019[1]
	UK / Republic of Ireland / Other Crown dependencies	24.6	
	Portugal	2.2	
	Latvia	1.5	
	Other Europe	2.9	
	Rest of World	3.9	
	Unknown	12.0	

Level of education	No formal qualification	15%	2018[3]
	High school	42%	
	Degree or professional qualification	43%	

Age and sex distribution of Guernsey population at March 2019[1]:

Age	Number			Percentage of population
	Female	Male	Total	
0-9	3,016	3,181	6,197	9.9
10-19	3,102	3,355	6,457	10.3
20-29	3,670	3,882	7,552	12.0
30-39	3,811	3,996	7,807	12.4
40-49	4,320	4,162	8,482	13.5
50-59	4,963	4,705	9,668	15.4
60-69	3,783	3,754	7,537	12.0
70-79	2,898	2,603	5,501	8.8
80-89	1,646	1,217	2,863	4.6
90+	455	187	642	1.0
Total	31,664	31,042	62,706	

References:

[1] Guernsey Facts and Figures 2020 (2020) States of Guernsey Data and Analysis, https://www.gov.gg/CHttpHandler.ashx?id=131184&p=0 Accessed: 17.8.21

[2] Guernsey Annual GVA and GDP Bulletin 2019 (First Estimates) (2020) States of Guernsey Data and Analysis, https://www.gov.gg/CHttpHandler.ashx?id=133037&p=0#:~:text=Guernsey%20GDP%20per%20capita%20in,GVA%20per%20capita%20in%202019.&text=The%20largest%20two%20components%20of,surplus%2C%20which%20represented%2041%25 Accessed: 17.8.21

[3] Jeffries L and Guille L (2019) Guernsey and Alderney Wellbeing Survey 2018, States of Guernsey Public Health Services. https://www.gov.gg/CHttpHandler.ashx?id=122310&p=0 Accessed 17.8.21.

Authors' position statement

Dr Martin Rabey

Dr Martin Rabey is a Specialist Musculoskeletal Physiotherapist (As awarded by the Australian College of Physiotherapists in 2009). He is an Advanced Scope Physiotherapist in the Spinal Surgery and Foot / Ankle Orthopaedic Departments of a tertiary teaching hospital in Perth, Western Australia. He is a lecturer at Curtin University, currently teaching anatomy and evidence-based practice. His research focusses on multidimensional interactions in musculoskeletal pain.

Prof. Helen Slater

Prof. Helen Slater is a Senior Clinical Researcher and Specialist Musculoskeletal Physiotherapist (As awarded by the Australian College of Physiotherapists in 2007). She co-leads a research group with Prof Andrew Briggs, at Curtin University with a focus on strategic capacity-building initiatives in musculoskeletal pain to strengthen health systems and re-orient health services to support consumers receiving 'right' pain care. She combines clinical pain research and health services research to support the implementation of musculoskeletal Models of Care. The enabling role for digital technologies (eHealth and mHealth) in supporting sustainable musculoskeletal Models of Care is a key focus.

Dr Clair Hebron

Dr Clair Hebron is a musculoskeletal physiotherapist and academic. She leads the MSc Musculoskeletal Physiotherapy course at the University of Brighton in the UK. Her research seeks to explore persons' experience of providing and seeking care and developing conceptual understanding and critical insights into practice (using a range of different methodologies including grounded theory, phenomenology, phenomenography and critical theory). She also has experience of quantitative research including investigation into the effects of mobilisation treatment to the lumbar spine.

Assoc. Prof. Niamh Moloney

Dr Niamh Moloney is a Musculoskeletal Physiotherapist and Associate Professor at Curtin University. Her teaching focuses on clinical pain science, specifically the integration of multi-dimensional pain assessment and treatment into clinical practice. Her research focuses on complex pain profiles including pain sensitivity and its influence on patient presentations, treatment responsiveness and outcomes. She also researches in the field of pain following breast cancer treatment.
